# Surface Acoustic Wave (SAW) Sensors: Physics, Materials, and Applications

**DOI:** 10.3390/s22030820

**Published:** 2022-01-21

**Authors:** Debdyuti Mandal, Sourav Banerjee

**Affiliations:** Integrated Material Assessment and Predictive Simulation Laboratory, University of South Carolina, Columbia, SC 29208, USA; dmandal@email.sc.edu

**Keywords:** surface acoustic waves (SAW), chemical sensors, point-of-care (POC), biosensors, SAW devices, SAW wave, lithium niobate, lithium tantalate, interdigitated electrodes, IDT, crystal cut, MEMS, ZnO, AlN, GaAs, GaN, Rayleigh wave, Love wave, Lamb wave, SH-wave

## Abstract

Surface acoustic waves (SAWs) are the guided waves that propagate along the top surface of a material with wave vectors orthogonal to the normal direction to the surface. Based on these waves, SAW sensors are conceptualized by employing piezoelectric crystals where the guided elastodynamic waves are generated through an electromechanical coupling. Electromechanical coupling in both active and passive modes is achieved by integrating interdigitated electrode transducers (IDT) with the piezoelectric crystals. Innovative meta-designs of the periodic IDTs define the functionality and application of SAW sensors. This review article presents the physics of guided surface acoustic waves and the piezoelectric materials used for designing SAW sensors. Then, how the piezoelectric materials and cuts could alter the functionality of the sensors is explained. The article summarizes a few key configurations of the electrodes and respective guidelines for generating different guided wave patterns such that new applications can be foreseen. Finally, the article explores the applications of SAW sensors and their progress in the fields of biomedical, microfluidics, chemical, and mechano-biological applications along with their crucial roles and potential plans for improvements in the long-term future in the field of science and technology.

## 1. Introduction

Human civilization is standing at a juncture in which the application of sensors in various fields is at its peak, which has never been seen before, and this will only increase in the future. Applications intensively rely on many devices and sensors that sense the environment, process information, and subsequently respond to the surroundings with the help of actuators. For daily actions, education, medical safety, and entertainment, sensors and actuators are key to the future. It is required that sensors and actuators should be responsive as quickly as possible and miniaturized at the same time. It is also realized that the sensors should be available at low cost for general affordability. These sensors and actuators are often called Microelectromechanical Systems (MEMS) [1,2,3,4,5]. MEMS in itself is a vast field, and it has applications in a wide variety of sectors such as electronics, aerospace, automotive, chemical, optical, wireless communications, biomedical, etc. The biosensor, which is a sub-field of the biomedical devices, is widely used for early diagnostics, and the detection of analytes now utilizes MEMS technology. A key aspect of using MEMS devices as biosensors is that they have made a promising impact on medical research. The devices have the potential to transform a complete wet laboratory into a tiny, miniaturized chip. They can even have wider applicability, from mechanical to chemical uses and in electrical and civil applications, along with many other fields [6,7,8,9,10]. These devices consist of both electrical and mechanical components under a single unit and can measure temperature, pressure, viscosity, stress, and mass change [11,12,13,14,15,16]. In the biomedical and chemical sectors, the lab-on-a-chip devices have attracted attention in recent decades. Advancements such as a reduced sample size, faster reaction and analysis, high-throughput, automation, and portability makes them highly demanded devices. However, irrespective of their field of application and advancements, and while lab-on-a-chip devices are very effective at sensing different parameters, they also suffer limitations. One of the major limitations comes from the requirements of simultaneous sensing and the actuation in an environment while engaging with mutually exclusive physics and mechanisms [17,18].

**Figure 1:**
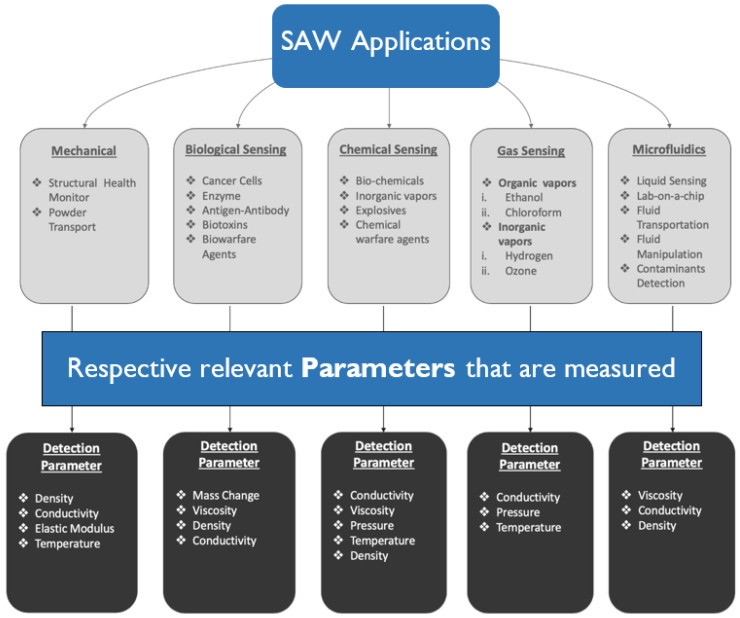
Classification of SAW applications and their detection parameters.

To overcome such limitations, acoustic wave devices are proposed for both sensing and actuation under a single platform simultaneously [19,20]. The physics of surface acoustic waves (SAWs) were found to be valuable for MEMS devices that require both sensing and actuation, generally known as SAW devices or sensors. SAW sensors may cover a wide range of applications, which are not only limited to sensors and actuators but also as filters, oscillators, transformers, etc. These applications were made possible by using piezoelectric components as the central backbone of the SAW sensors. Piezoelectric crystals have a unique property of electromechanical coupling that makes them remarkably robust [21,22,23]. Surface acoustic wave technology consists of a piezoelectric substrate and interdigitated electrodes or transducers (IDTs). The IDTs are patterned on the surface of the piezo substrates by design. Together, they transform the applied electrical energy into mechanical energy and generate surface acoustic waves. This process is known as actuation. The electrical component generates the mechanical energy as acoustic waves in the piezoelectric substrate under the dynamic change of the electrical input signal due to the electromechanical coupling [24,25,26,27,28]. The generated surface acoustic waves propagate on the surface of the piezoelectric wafer and interact with the other patterned interdigitated electrodes. The IDT at the receiving end produces electrical signals through the mechanoelectrical coupling of the piezoelectric substrate sensing the surface waves using the reverse mechanism. This process is referred to as acoustic sensing. The surface acoustic waves can be manipulated in various ways and can be modified considering the variation of different physical changes, which makes them magnificent actuators and sensors [29,30,31]. SAW devices are widely used in many fields, such as in mechanical, chemical, electrical, physical science, and biological applications. Figure 1 depicts the applications of SAW devices in various fields and their respective sub-fields. These sensors have the capability of detecting slightest disturbances on the surface [32,33,34,35,36,37,38,39,40,41,42,43,44,45,46,47,48,49]. The acoustic wave technology is very useful in the sensing and structural health monitoring of mechanical structures. Studies have shown that SAW devices are often used as actuators for repairing or quarantining the damage in a mechanical system, which is crucial in structural health monitoring [50]. SAW devices have been successfully demonstrated for material transport in micro and nanoscales [51]. SAW-based technology is gradually influencing the field of biosensing day-by-day. Applications from the detection of cancer cells, DNA, antigens–antibodies, biotoxins, and the detection of biowarfare agents, etc. to bio-actuation and biofluid transport have been successfully demonstrated using acoustic wave devices [52,53,54,55,56,57,58,59]. In addition to the field of biosensing, SAWs have made a significant impact on chemical and gas sensing. This includes the detection of chemical warfare agents, explosives, organic and inorganic vapors, etc. [60,61,62,63,64,65,66,67,68]. The bio-MEMS devices that are frequently used in the field of biomedical applications have transformed lab-on-a-chip research. Organ-on-a-chip or lab-on-a-chip devices utilize the physics of the microfluidic system. SAW sensors have gained a great deal of attention in the area of microfluidics in the past few years. Only recently, the transportation and control of cells and fluids in micro-nano scales, sensing of specific fluid species, sensing of heavy metals, and detection of contaminants in the bio environment have been proposed using SAW-based microfluidic devices [69,70,71,72,73,74,75,76,77]. SAW sensors have been widely utilized recently in light sensing as well. The sensing area of the SAW devices is coated with a photosensitive chemical that changes its properties upon light exposure, resulting in the mass loading effect, which changes the acoustic velocity and subsequently affects the frequency shift utilized for sensing purposes [78,79,80]. In a very similar manner, SAW devices are also widely being used as pH sensors [81,82].

Over the years, many studies and reviews have been conducted on SAWs and devices. Go et al. (2017) [17] conducted a review on SAW devices for chemical and microfluidics sensing, Länge et al. (2019) [83] published a review on bulk and SAW sensor arrays for multi-analyte detection. Similarly, Devkota et al. (2017) [84] published an article on SAW sensors on chemical vapors and gases. Gowdhaman et al. (2018) [85] conducted a study of SAW sensors for the detection and identification of toxic gases. Additionally, Priya et al. (2015) [86] conducted a short review on SAW sensors. Likewise, many other authors and researchers have studied and conducted reviews on SAW sensors and their various applications in different fields. None of the above review articles describes an integrated approach with the physics of waves, their relevance to the selection of piezoelectric substrate, substrate cut and wave directions, the physics of designing the interdigitated electrodes, and how different wave physics and different materials could be used for different applications under one umbrella. In this article, we present four pillars of SAW sensors—a. the physics of waves, b. the physics of piezoelectric materials and their cut and their influence on wave propagation, c. the physics of electrode design, and d. fundamental physical variables—that could be sensed by exploiting the wave physics in an integrated form.

In SAW devices, a piezoelectric substrate is used that generates surface acoustic waves when an electrical voltage is applied. In other words, the acoustic devices are responsive to the physical parameters of the species that interact with the mechanical properties of the guided waves in the SAW sensors. These interactions are manifested by altering the respective electrical output from the device in the form of current, voltage, or capacitance. As a result, SAW sensors that are used for chemical or biological sensing are designed in such a way that the transducer layer should have the opportunity to interact with the chemical or biological agents. Then, these interactions are quantitatively converted into a desired mechanical or electrical response as output. Hoummady et al. (1997) described how the acoustic phase velocity can be affected by various physical parameters [87,88,89]. In a linear sense, Equation (1) elucidates the interruption of the acoustic wave velocity by mass, electrical, mechanical, and environmental properties. Let us assume that V is the velocity of the SAW in the substrate and is a function of multiple physical and environmental parameters. For example, V is a function of mass (m); the effect of electrical properties (E)—e.g., conductivity; the effect of mechanical properties ($M_{i}$)—e.g., elastic modulus and viscosity; and the effect of the environment ($e_{v}$), which may include temperature, pressure, and humidity. Further, if ∂V is the change or perturbation of the wave velocity, then following the chain rule, dV can be expressed as follows.

(1) $$dV=\left(\frac{\partial V}{\partial m}\;dm+\frac{\partial V}{\partial E}\;dE+\frac{\partial V}{\partial M_{i}}\;dM_{i}+\frac{\partial V}{\partial e_{v}\;}\;de_{v}\right)$$

**Figure 2:**
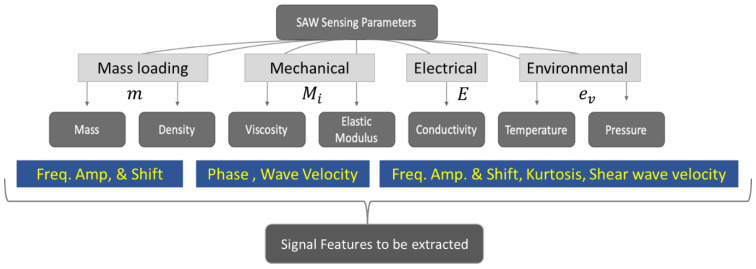
Classification of the SAW sensing parameters.

Surface acoustic devices explore various sensing parameters. It is evident from the previous flowchart that, irrespective of their applications, SAW sensing parameters can be broadly classified into few parameters such as mass, density, viscosity, elastic modulus, conductivity, temperature, and pressure. Equation (1) collectively present the effects of these parameters on the wave velocity. It is apparent that the collective change in wave velocity would not be able to distinguish the change in individual variables in Equation (1). However, using SAW sensors, measuring the change in wave velocity (dV) is not the only method to measure the influence parameters or the sensing variables. Using SAW sensor, one could collect an entire time history signal from an IDT receptor, as discussed later in this article. The wave signals received by the receptor end the IDTs could be further analyzed using different analysis methods. The wave signals carry various signal features that are possibly influenced by the individual sensing variable but appear to be coupled while measuring the *dV*. Hence, measuring the change in amplitude of the central frequency, the frequency shift of the given frequency input, kurtosis, change in phase, and higher-order frequency peaks to measure nonlinearity will help us to measure the change in sensing variables individually. Figure 2 describes the classification of different sensing parameters that are mostly exploited in SAW sensors and possible wave features to find their effects.

Here, it is necessary to discuss the sensitivity and selectivity of the SAW sensors. In the most basic terms, sensitivity defines how small of a change in a sensing parameter could be detected by a sensor. Sensitivity of detection is defined based on the detection of the main analytes, not the parameters in Figure 2 directly. Please note that the sensing parameters in Figure 2 may be the secondary parameters because of external analytes or some other form of mechanism. It is possible to have a higher sensitivity for a specifically designed SAW sensor to measure the change in temperature but have very poor sensitivity when measuring the mass loading. Hence, SAW sensors are not certain to have high sensitivity for all secondary parameters. Targeted application-driven SAW sensors must be designed with a specific geometric configuration and specific material, with a specific design of IDTs to ensure the detection of a specific mechanism through a specific secondary parameter. For example, a layer of antigen (Ag) will bind with a specific antibody (Ab). This physical mechanism can be implemented on a SAW sensor, and if the Ab combines with the Ag, the mass loading will change compared to a layer of a bare Ag. This change in mass loading may affect the wave velocity and/or the frequency amplitude. The targeted detection of the change in the wave velocity (feature) will point to the detection of the mechanism of Ab–Ag combination (primary) via the change in mass loading (secondary). The ultimate target here will be the detection of an Ab. Thus, sensitivity could be defined by how small an amount of Ab could be detected. In a nutshell, a feature points to the change in the secondary parameters as observables to detect the primary mechanism to be detected. On the other hand, selectivity defines how specific the detection is. For example, in the above experiment, if a different antibody (Ab) is given and the Ab does not combine with the antigen (Ag), then the mass loading will not increase, and the detection should be negative. Detection should be positive and only positive when the Ab is present but should be negative and only negative when Bb is present. On many occasions, SAW sensors may provide false positives or false negatives. Both situations compromise the sensitivity and selectivity of the sensors. When selectivity is confirmed, it is not guaranteed to obtain a highly sensitive sensor. Vice versa, if sensitivity is enhanced, it is not guaranteed to have a highly selective sensor. Thus, in the author’s opinion, it is necessary to impose multichannel and multi-aperture detection to simultaneously ensure sensitivity and selectivity in SAW sensors. A higher number of fingers in IDTs and more than one IDT terminal for sensing may increase both the sensitivity and selectivity of the sensors. In the future, more innovative design of SAW sensors will be required to achieve both of these aims simultaneously. The following sections shed light on this topic with a more detailed understanding and citing applications.

## 2. Types of Waves in SAW Devices

SAW devices are designed to operate at ultrasonic frequencies, and an ultrasonic wave propagates in the substrate. To design a specific SAW sensor for a specific application (based on target application), it is necessary to know different types of waves that can be generated in a substrate. In this section, different types of waves that are important for SAW devices are discussed.

### 2.1. Rayleigh Waves

**Figure 3:**
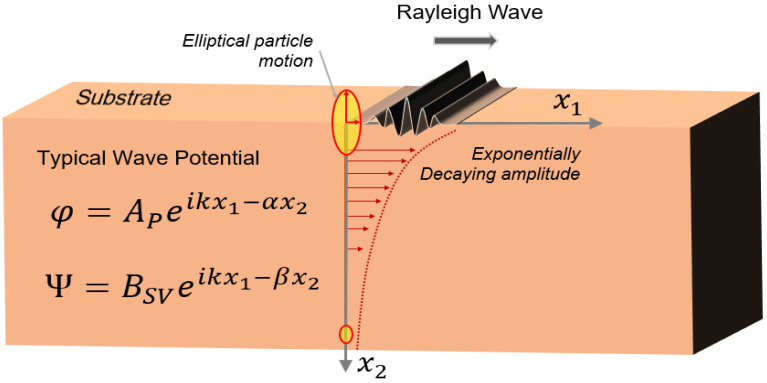
Schematic of the Rayleigh waves propagating through a substrate [91].

Rayleigh waves propagate through the surface of a substrate. The polarization of the Rayleigh waves is along the plane perpendicular to the surface of the substrate, and the amplitude of the particle motion decreases exponentially with the depth of the substrate. Rayleigh waves are the modal superposition of longitudinal (P) and shear vertical (SV) wave components with traction-free boundary conditions on the substrate [24]. Due to the property of this wave, the surface particles move in an elliptical fashion, normal to the surface and parallel to the direction of the propagation [90,91]. In this type of wave, the velocity of the wave is solely dependent on the material of the substrate and the orientation of the crystals. The Rayleigh wave velocity is defined by the following equation [91].

(2) $$\;c_{R}=c_{S}\left(\frac{0.87+1.12\nu }{1+\nu }\right)\;\;$$

where ν is the Poisson’s ratio and $c_{S}$ is the shear wave speed through the substrate. Figure 3 shows the schematic of the Rayleigh waves propagating through a surface.

The effective penetration of the Rayleigh waves is usually less than a wavelength along the direction into the substrate. The longitudinal and shear potentials that are assumed to solve the Rayleigh wave are shown in Figure 3, where two unknowns to describe the longitudinal wave amplitude ($A_{P}$) and shear-vertical wave amplitude ($B_{SV}$) are assumed. A detailed derivation of the Rayleigh wave–particle motions can be found elsewhere [91]; however, final equations for the particle motion in a Rayleigh wave are given by the following equations:

(3) $$u_{1}\left(x_{2}\right)=Ai\;\left(k.\exp\left(-\alpha x_{2}\right)\;–\;\frac{\left(\beta^{2}+k^{2}\right)}{2k}\exp\left(-\beta x_{2}\right)\right)$$

(4) $$u_{2}\left(x_{2}\right)=A\;\left(-\alpha .\exp\left(-\alpha x_{2}\right)+i\;\frac{\left(\beta^{2}+k^{2}\right)}{2\beta }\exp\left(-\beta x_{2}\right)\right)$$

where k is the wavenumber along the direction of the wave propagation along the $x_{1}$ axis; $\alpha =\sqrt{k^{2}-\frac{\omega^{2}}{c_{p}^{2}}}$ and $\beta =\sqrt{k^{2}-\frac{\omega^{2}}{c_{s}^{2}}}$ are the wave numbers along the $x_{2}$ axis for longitudinal and shear-vertical waves, respectively; $c_{S}$ is the shear wave speed; $c_{P}$ is the longitudinal wave speed in the substrate; and ω is the frequency of the wave.

### 2.2. Shear Horizontal Waves

**Figure 4:**
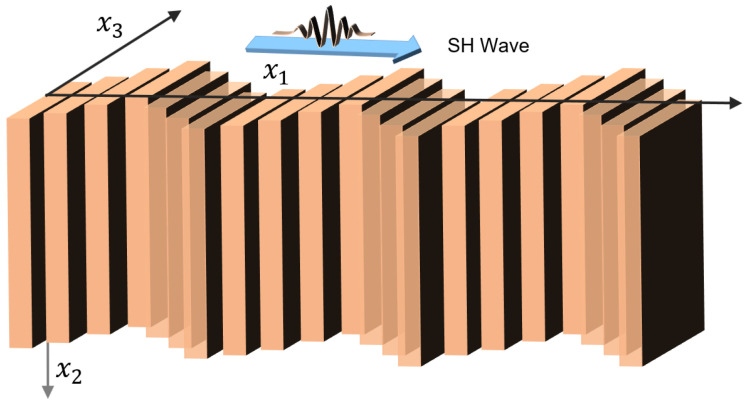
Schematic of the shear horizontal (SH) waves propagating through a substrate [91].

For shear horizontal (SH) waves, the motion of the particles is perpendicular (Figure 4) to the direction of the wave propagation [91]. However, unlike the Rayleigh wave, the amplitude of the particle displacements does not decay exponentially along the depth. As the amplitude does not decay, multiple wavelengths are feasible to transmit though the substrate, and hence this type of wave travels several wavelengths into the substrate or saturates the complete thickness of the substrate used. This makes it sensitive to the changes in the device surface. The shear horizontal-SAW sensors are very useful and are widely used for biochemical detection, especially in liquid media. Compared to the bulk waves, the surface waves are more sensitive to the perturbations generated in the environment. The SH-SAW device can be scaled to work at more than a ~100 MHz frequency. This enables the device to have higher sensitivity while keeping a low signal-to-noise ratio. These devices are not only small and robust but also are easy to incorporate into on-line, low-cost systems [92]. The sensitivity of the SH-SAW devices is often enhanced using a thin guiding layer. Different dielectric materials such as silicon dioxide, silicon nitride, and various polymers such as PMMA and polyimides are used as the guiding layers on SH-SAW devices. With the help of these guiding layers, the propagation velocities are reduced, and the acoustic energies are trapped in the vicinity of the sensing surface, thus enhancing the sensitivity to the surface perturbations. The biological/chemical detection process using the SH-SAW uses the delay line configurations where the sensing area is between the two IDTs. To design these devices, the effect of the sensor-side IDT is accounted for in the design responses. Equation (5) states the sensor response that can be modeled by the transfer function [93].

(5) $$T_{2}\left(f\right)=T_{12}\left(f\right)e^{-i\left(2\pi l/\lambda \right)}e^{i\left(2\pi \delta ls/\lambda \right)\;}\;e^{-als}$$

where $T_{2}$ and $T_{12}$ are the transfer functions in 2-2 and 1-2 directions, δ is the fractional velocity change of the SH-SAW due to the sensing effect, a is the attenuation coefficient due to the wave guide layer and the bio/chemically sensitive layer composite, and l and ls are the IDT’s center-to-center separation and sensing path length.

The waveguiding of the guiding layer of an appropriate thickness occurs when the shear wave velocity in the guiding layer is less than the velocity in the substrate. By assuming that the SH wave is coupled to the IDT, the fractional change in the wave velocity and the measure of the sensitivity to the mechanical perturbations on the surface are obtained using perturbation theory [93]:

(6) $$\frac{\Delta V}{V}=-\frac{V_{SH}}{4}\;\left(\rho h\right)\;\left[1-{\left(\frac{V_{M}}{V_{SH}}\right)}^{2}\right]{\left|U_{2}\right|}^{2}$$

where $V_{SH}$ is the unperturbed velocity in the substrate, $V_{M}\;$ is the velocity in the guiding layer, ρ is the mass density, h is the layer thickness, and $U_{2}$ is the normalized particle velocity displacement amplitude at the surface. In the case when the resulting wave does not have dispersion and the sensing path equals the propagation length of the SH-SAW, the equation above also describes the relative frequency shift. Due to this feature, assuming a purely elastic film, the sensitivities due to mass loading and viscoelastic loadings are calculated by

(7) $$S_{m}=\underset{\Delta m\to 0}{\lim}\left(\frac{\Delta f}{f}\right)/\Delta m\;\;$$

where Δm=ρh. The change in the Δf  is mostly contributed by the mass loading, but at times the viscoelastic loadings contribute to this change as well. In biosensing and other chemical applications, the above equation is more than suitable because the biolayer consisting of the bioreceptors on top of the guiding layer used for detection or capturing the bio-analytes is on the order of a few molecules and consists of negligible viscoelastic contributions. This phenomenon is the reason why many researchers have used the frequency shifts of SH-SAW devices for acoustic biosensing/chemical applications. Examples of materials that can generate SH-SAW waves include 36° YX-cut LiTaO_3_, Quartz, 36° YX-cut LiNbO_3,_ and 64° YX-cut LiNbO_3_. Please refer to Section 3.2 for an understanding of crystal-cut SAW wave propagation. A SAW sensor based on SH waves can be used for liquid and gas sensing [90]. Figure 4 shows the schematic of the shear horizontal waves.

The displacement function for the SH wave can be written as

(8) $$\;\;\;\;\;\;u_{3}\left(x_{1},x_{2}\right)=\left(\exp\left(ikx_{1}-i\beta x_{2}\right)+R\exp\left(ikx_{1}+i\beta x_{2}\right)\right)$$

SH wave shear stress along direction $x_{3}$ but perpendicular to $x_{1}$ is the only non-vanishing stress that acts in the substrate boundary and can be expressed as

(9) $$\;\;\;\;\;\;\sigma_{13}=2ikc_{s}^{2}\rho \;\cos\left(\beta x_{2}\right)\exp\left(ikx_{1}\right)$$

where k is the wavenumber along the direction of the wave propagation along the $x_{1}$ axis, $\beta^{2}=k^{2}-\frac{\omega^{2}}{c_{s}^{2}}$, $c_{S}$ is the shear wave speed, and ρ is the density of the substrate.

### 2.3. Lamb Waves

**Figure 5:**
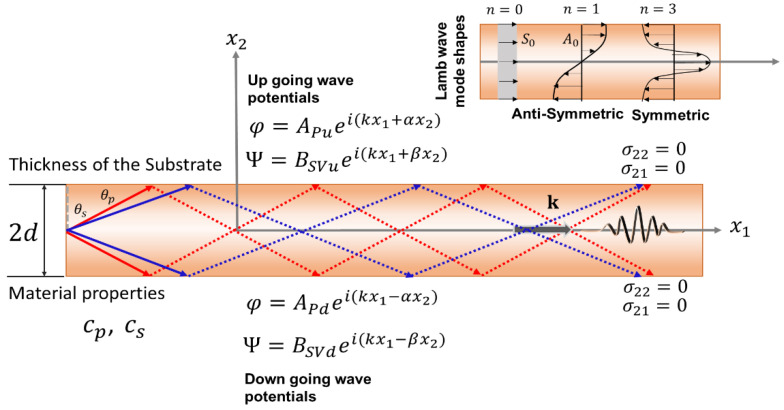
Schematic of the Lamb waves propagating through a substrate. Mathematical equations are those used in deriving the Lamb wave dispersion [91].

Lamb waves are generated in a wave guide between two parallel surfaces; for example, between the upper and lower surfaces of a wafer or substrate. In other words, Lamb wave saturates the complete thickness of the substrate. Lamb waves are categorized into two modal wave types—antisymmetric and symmetric waves—that can propagate through the plate independently depending on the frequency of the wave. The Lamb wave velocity is dependent on the product of the frequency and the wafer thickness. The velocity of each Lamb wave mode varies with frequencies because they are highly dispersive. Depending on the media and excitation frequency, Lamb waves can propagate at very high velocities ranging from 800 m/s to 6000 m/s, making them suitable for SAW applications. The Lamb wave frequency of propagation increases with increasing thickness. Hence, for sensor applications using Lamb waves, either it is necessary to use a lower frequency (below 5 MHz) or use incredibly thin wafers to access higher frequencies. Figure 5 represents the schematic of the symmetric and antisymmetric Lamb waves [91].

In Figure 5, two sets of waves are presented: first, the upward longitudinal and shear vertical waves, and second the downward longitudinal and shear vertical waves. In Figure 5, four unknowns are assumed to describe the upward longitudinal wave amplitude ($A_{Pu}$) and shear-vertical wave amplitude ($B_{SVu}$), and downward longitudinal wave amplitude ($A_{Pd}$) and shear-vertical wave amplitude ($B_{SVd}$). Additionally, k is the wavenumber along the direction of the wave propagation along the $x_{1}$ axis, and $\alpha =\sqrt{k^{2}-\frac{\omega^{2}}{c_{p}^{2}}}$ and $\beta =\sqrt{k^{2}-\frac{\omega^{2}}{c_{s}^{2}}}$ are the wave numbers along the $x_{2}$ axis for longitudinal and shear-vertical waves, respectively; $c_{S}$ is the shear wave speed; $c_{P}$ is the longitudinal wave speed in the substrate; and ω is the frequency of the wave.

Lamb waves consist of antisymmetric (A) and symmetric (S) wave modes. In the symmetric Lamb wave mode, the displacements of the particles are given by the following equations:

(10) $$u_{1}\left(x_{2}\right)=Ai\;k\cdot \cos\left(px_{2}\right)+Bi\;q\cdot \cos\left(qx_{2}\right)$$

(11) $$\;u_{2}\left(x_{2}\right)=-Ap\cdot \sin\left(px_{2}\right)-Bi\;k\cdot \sin\left(qx_{2}\right)$$

where $p^{2}=\frac{\omega^{2}}{c_{p}^{2}}-k^{2}$ and $q^{2}=\frac{\omega^{2}}{c_{s}^{2}}-k^{2}$, synonymous to α and β but imaginary.

### 2.4. Love Waves

**Figure 6:**
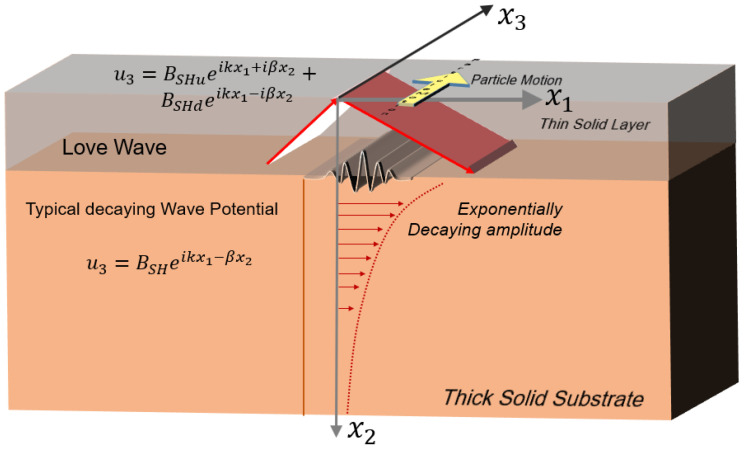
Schematic and mathematics of the Love waves propagating through a surface [91].

Like Rayleigh wave, an SH wave with a decaying amplitude along its depth is not sustainable. Hence, a thin layer is necessary to guide the SH wave, and then the wave can decay along with the depth of another layer attached to the thin layer. Such SH waves are called Love waves. Hence, the Love waves are generated at the interface of two solid elastic substrate layers where one layer is very thick and the other layer is a thin part on top of the thick layer. These are the guided waves that propagate through a thin layer of deposition made on top of the SAW substrate. Very high acoustic energies are concentrated in the thin guiding layer when Love waves are generated. Thus, these waves are very sensitive to mass loading on the substrate. Substrates such as 36° YX-cut LiTaO_3_ and 64° YX-cut LiNbO_3_ are suitable for Love waves where, on top of the SAW substrate, guiding layers such as SiO_2_, ZnO, PMMA, SU-8 photoresist, and TiO_2_ are deposited. This type of wave has the advantage of having the highest sensitivity (amongst the SAW sensors) due to its concentrated guiding characteristics. Furthermore, due to their surface-based nature, Love waves can propagate into liquid media, making them suitable for biosensing. Figure 6 represents the schematic of the Love waves. The motion of the particles of the Love wave are orthogonal to the direction of the wave propagation [91].

In Figure 6 three sets are waves are presented: first, an upward shear horizontal wave; second, a downward shear horizontal wave in the thin solid layer; and third, a decaying shear horizontal wave. In Figure 6, three unknowns are assumed to describe the upward shear-horizontal wave amplitude ($B_{SHu}$), downward shear-horizontal wave amplitude ($B_{SHd}$) in the thin solid layer, and a decaying shear-horizontal wave amplitude ($B_{SH}$) in the thick solid layer. Additionally, k is the wavenumber along the direction of the wave propagation along $x_{1}$ axis, $\beta =\sqrt{k^{2}-\frac{\omega^{2}}{c_{s}^{2}}}$ is the wave numbers along the $x_{2}$ axis for the shear-horizontal wave, $c_{S}$ is the shear wave speed in the substrate, and ω is the frequency of the Love wave.

Below, Table 1 summarizes different types of waves discussed above and materials to generate them, application frequencies, attenuation factors and miscellaneous comments are also presented. 

## 3. Materials for the SAW Devices

### 3.1. Constitutive Properties of Piezoelectric Materials

SAW technology is truly based on the piezoelectric phenomenon. Piezoelectric crystals have fixed dipoles that re-orient in a fixed direction with the application of an external electric field to the crystal. This re-orientation of the dipoles causes mechanical strains in the piezo-crystals. The piezoelectric effect works exclusively with the anisotropic crystal lattice. Hence, high importance is given to the cut and the orientation of the crystal during the design and manufacturing of the base piezoelectric material. The most important physics that separate piezoelectric devices from others is their unique property of electromechanical coupling. Unlike pure mechanical or electrical devices, the electromechanical property of the piezoelectric crystals enables an electrical response with mechanical perturbation and vice versa. [94,95,96,97,98,99,100]. The following are the constitutive equations that describe the piezoelectric phenomena [101]:

(12) $$\begin{array}{cc} \mathrm{Strain}–\mathrm{Charge Form}: & \mathrm{Strain}–\mathrm{Charge Form}:\\ S=s_{E}\cdot T+d^{t}\cdot E\; & T=c_{E}\cdot S-e^{t}\cdot E\\ D=d\cdot T+\varepsilon_{T}\cdot E & \;D=e\;\cdot \;S+\varepsilon_{S}\cdot E\\ \mathrm{provided},\;c_{E}=s_{E}^{-1};\;e=d\cdot s_{E}^{-1}\;\mathrm{and} & \;\varepsilon_{S}=\varepsilon_{T}-d\cdot s_{E}^{-1}\cdot d^{t} \end{array}$$

where **S** denotes the vectorized strain tensor; T  represents the vectorized stress tensor; ε refers to the dielectric permittivity tensor; E  refers to the external electric field; **s** represents the compliance matrix, written in 6 × 6 matrix form, where it is a fourth-order tensor; **D** denotes the electric displacement; and d is a third-order tensor representing the piezoelectric constant, written in a 3 × 6 matrix to be multiplied with the vectorized stress tensor. The governing differential equation of wave propagation in the substrate takes the traditional Navier’s equation as follows:

(13) $$\;\rho \frac{\partial^{2}u_{i}}{\partial t^{2}}=\frac{\partial \sigma_{ij}}{\partial x_{j}}+f_{i}$$

where $u_{i}$ is the displacement of the media in three directions, $\sigma_{ij}$ is a stress tensor, and $f_{i}$ is the body force in the *i*-th direction. The solution of the above equation with an appropriate form of the constitutive relation of the substrate and boundary condition will provide the different forms of waves discussed in the previous section. Specific solutions of SAW with different scenarios are not presented herein and can be found elsewhere [91]. However, the displacement potential functions that are required to solve Equation (13) for different wave types are given in Section 2 (Figure 3, Figure 4, Figure 5 and Figure 6).

Additionally, specific to the substrate, an important factor that determines the efficiency of a piezoelectric material is the piezoelectric coupling coefficient factor. This is also at times referred to as the electromechanical coupling coefficient, which is also defined as the ratio of the mechanical energy collected in return to an electrical input or vice-versa. The electromechanical coupling coefficient is given by *k,* where

(14) $$k=\frac{d}{\sqrt{s\varepsilon }}$$

**Figure 7:**
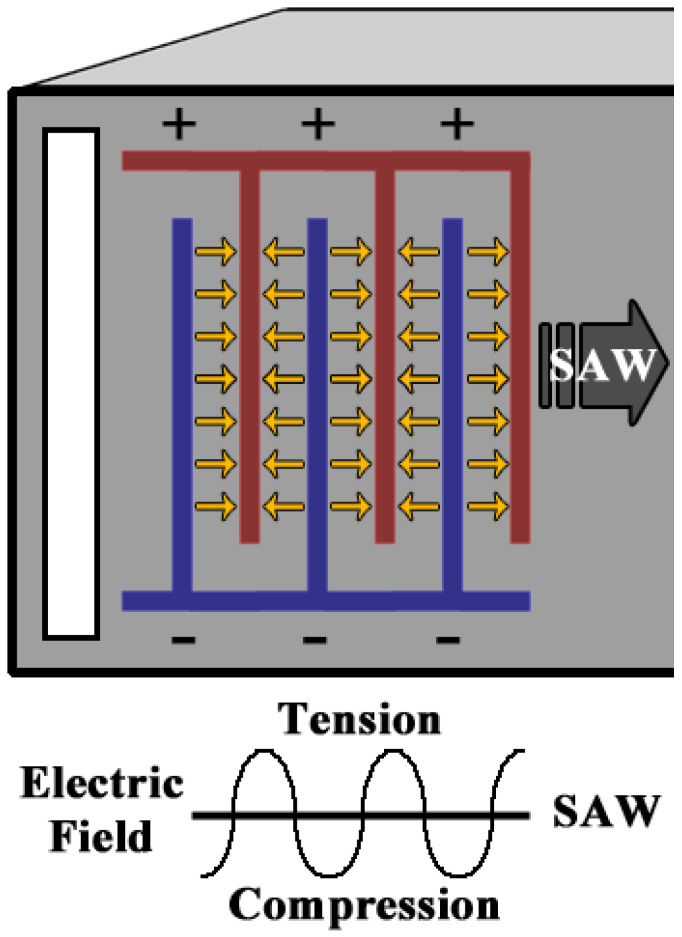
SAW generation phenomena in piezoelectric wafer [4].

To receive maximum efficacy of the piezoelectric material, a higher value of the piezoelectric charge constant is required. A SAW device consists of interdigitated electrodes (IDTs) patterned on top of the piezoelectric wafer. The input terminals of the IDTs are connected to an alternating current (AC) terminal (sinusoidal electric signal). As a result, the polarity of the interdigitated electrodes is alternated with the AC signal, which in turn generates alternating positive and negative electric fields between the fingers of the IDTs. Due to the piezoelectric properties, these alternating regions induce mechanical strains; i.e., local tension and compression produced between the fingers of the electrodes. This phenomenon generates mechanical waves—a sinusoidal effect along the surface of the wafer. As per the conventional setup of the device, the mechanical waves are generated along both sides of the electrodes, thus producing half of the energy of the waveform propagating through the delay line towards the direction of the output IDTs [102,103,104]. Figure 7 shows a basic schematic of the phenomenon of a surface acoustic wave generated in a piezoelectric wafer.

The surface acoustic wave propagates through the delay line to another set of the interdigitated electrodes on the other side. Due to the direct piezoelectric property, the surface acoustic wave (crests and troughs) produces an alternating electrical output signal when it interacts with the IDTs on the opposite side. The output signal is recorded, compared, and analyzed with the input signal. The changes in the wave velocity and the amplitude recorded from the sensing terminal can be determined. A frequency shift, or the amplitude of a specific frequency, and the time-delay of the output signal are used to measure different physical parameters and properties [105,106,107,108]. The material selection of the substrate or the wafer equally plays a crucial role in the design of SAW devices. The substrate selection requires both the material type and considerations of crystal orientations for the desired piezoelectric output. The properties that are highly involved in the material selection include the electromechanical coupling factor, coefficient of thermal expansion, wave velocity, compatibility, and finally the cost of the raw materials. The wave velocity through the material is crucial in the design of SAW devices. The crystalline cut of the piezo materials in a desired orientation enhances the electromechanical coupling of the piezoelectric wafer, which is highly important for the design [109,110,111,112]. Additionally, a low coefficient of thermal expansion of the piezoelectric materials is preferred, excluding in temperature sensing using SAW devices. Table 2 shows the comparison of various piezoelectric substrates with respect to the orientation, wave velocity, electromechanical coupling, and thermal coefficient factors.

### 3.2. Piezoelectric Crystal Cuts and Their SAW Propagation

**Figure 8:**
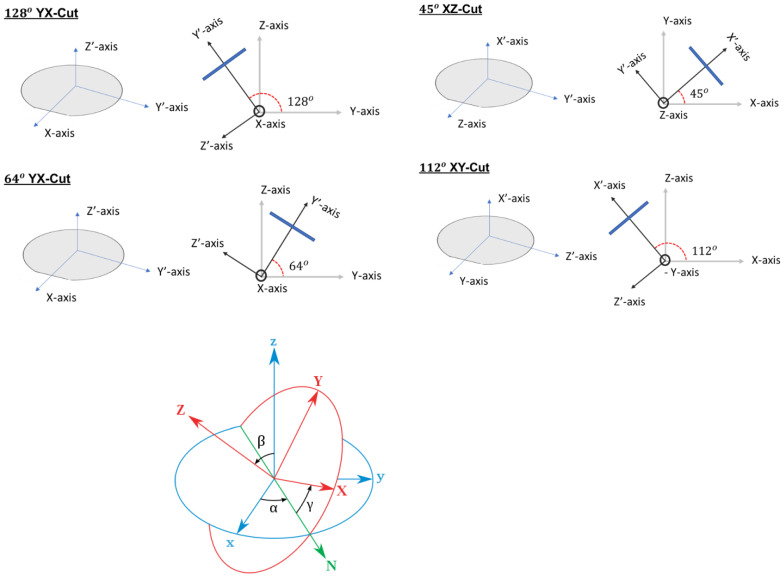
Different orientational cuts of the Lithium Niobate crystal (**top**) and the Eulerian angles (**bottom**).

Although there are very wide varieties of piezoelectric crystals, these crystals are often cut to a specific orientation to achieve the highest piezoelectric efficiency and also for a particular type of wave to be generated based on the user’s requirement. For example, 36° YX cut lithium tantalate generates shear horizontal waves. In a similar manner, a 128° YX cut of lithium niobate is capable of generating Rayleigh waves. A strong piezoelectric effect is often achieved using the crystal cuts. The values of the piezoelectric coefficient in the matrices have direct effects on the crystal cuts because of their effect on the electromechanical coupling coefficient, which subsequently corresponds to the efficacy of the piezoelectric material or substrate. The understanding of the nomenclature of a specific cut corresponding to a piezoelectric crystal is crucial for generating a particular type of wave and enhancing its piezoelectric effect. The first letter corresponding to a piezo-crystal cut refers to the thickness of the wafer along that direction, and the second letter corresponds to the rotation about that axis corresponding to a specified angle. For example, a 64° YX lithium niobate wafer has the thickness of the wafer along the first letter, which is the Y axis, and then the crystal is rotated to 64° about the second letter, which is the X axis, and then the crystal is cut into wafers. Figure 8 shows the concepts of 128° YX and 64° YX cut lithium niobate crystal, 45° XZ cut Lithium Tetraborate, and 112° XY cut Lithium Tantalate.

The orientational cut of a particular piezoelectric crystal also has its effect on the wave velocity. A Lithium Niobate crystal without any orientational cut has a wave velocity of 3488 m/s, whereas a 128° YX cut Lithium Niobate crystal has a Rayleigh wave velocity of 3979 m/s. As mentioned above, a piezoelectric crystal is orientated and cut to specific angles and axes based on the types of wave generation and velocity requirement, but the orientational rotation also leads to the formation of three new different axes, namely x, y, and z, respectively. The transformed axes when compared to the original axes are orientated in certain angles known as the Eulerian angles. The Eulerian angles are represented by α, β, and γ, respectively. These angles are utilized in the fabrication of the piezoelectric wafers and are often used for the simulation process in predefining the axes of the plane of the piezo substrate. Based on these factors, we demonstrate a table (Table 3) with different piezoelectric crystals and their orientational cuts in generating different types of waves.

In the field of SAW technologies, not only the bulk substrates but also the thin film acoustic wave have attracted the attention of researchers over the past few years. Thin-film piezoelectric materials such as ZnO, AlN, and PZT (Piezoceramics) are regarded as one of the outstanding technologies for lab-on-a-chip and acoustofluidics devices [113,114,115]. The bulk substrates are often expensive and brittle. In such scenarios, the thin-film technologies have an advantage over them. Additional merits of thin-film technologies, such as their flexibility and the ability of depositing the film focused only into the acoustic sensing area irrespective of the whole substrate, save both time and cost directly [116,117]. In this process, the wave is propagated along the substrate without the requirement of the piezoelectric top layer. Amongst the thin-film piezoelectric materials, piezoceramics have been reported to demonstrate the largest piezoelectric constant and electromechanical coupling, which are both crucial for sensing and actuation, although PZT suffers some disadvantages such as high energy loss, low wave velocity, low-quality factors, and sometimes being difficult to fabricate [118,119]. ZnO is another thin-film material that is highly regarded for piezoelectric devices because of its higher piezoelectric coupling coefficient. It is reported to have low film stress and has better adhesion than most of the substrates, due to which thick films (few microns) can be deposited. In other words, ZnO films are acceptable for thick film surface acoustic wave devices. ZnO film-based SAW devices are regarded as very biocompatible, due to which they are popular and widely used in the field of biosensing [120,121,122,123,124,125]. Aluminum nitride has also attracted attention in the past few years. It is also considered to be one of the piezoelectric thin-film materials that can tolerate high temperature, making it quite useful for high temperature-based SAW applications. One of the most unique features about AlN films is that they have the highest wave velocity among all other thin film piezoelectric deposits, positioning the film as a popular thin-film piezoelectric material [126,127,128,129,130,131,132]. However, unlike the ZnO film, AlN cannot be used for thick films and hence is mostly used in the applications of thin films and high-frequency-based applications. Additional to these, certain materials such as gallium arsenide (GaAs), polyvinylidene fluoride (PVDF), and gallium nitride (GaN) are also excellent piezoelectric materials that are reported to be widely used in SAW devices. Only recently, aluminum gallium nitride (AlGaN) has also been experimented with as a potential piezo thin film [126]. Table 4 shows the material properties of various deposited piezoelectric materials used in surface acoustic wave devices.

## 4. Configuration of the Interdigitated Electrodes in the SAW Devices

Surface acoustic waves (SAWs) are basically electromechanical waves generated on the surface of a piezoelectric substrate. As discussed earlier, a traveling surface wave is directed along the crystal generated by the interdigitated electrodes utilizing the electromechanical coupling between the input electrical signal and the piezoelectric substrate. The interdigitated electrodes can be designed in numerous ways and in a different configuration depending on the target applications and user requirement. Additionally, efforts were made towards achieving the valuable and unique design of IDTs to reduce the insertion loss and achieve high center frequency responses. The frequency bandwidth and the electrical impedance are highly dependent on certain parameters such as the electrode width, spacing, aperture, and number of fingers of the designed IDT. Let us call the IDTs transducers for the SAW sensors. Transducers are designed for electromechanical transduction and should be designed based on the amount of energy to be transmitted at certain frequency. Through electromechanical transduction, mechanical energy as a stress wave propagates in the substrate. The amplitude, phase, and frequency of the stress wave to be generated in the substrate depend on the transducers. Once the wave is generated, the reflected wave energy also travels back into the substrate in the opposite direction, which may destructively interfere with and diminish the response signal. Hence, the design of IDTs or transducers should be such that reflection by the IDTs could be reduced to increase the sensitivity of the SAW sensors. On the other hand, to enhance both sensitivity and selectivity, a high frequency wave is required in the same sensing platform. This could be achieved by generating the higher harmonics of the central frequency of excitation. The generation of a central frequency through transduction, the reduction of reflection, re-generation, and accessing the higher harmonics is the guiding factor of the design of IDTs. These properties could be manipulated using the following electrode parameters: electrode width (w), spacing (s), aperture (W), number of fingers (N), and apodization. Few physics facts are noted. A higher electrode width (w) causes a lower central frequency f to be generated. A higher spacing (s) causes lower refection; however, spacing (s) and width (w) should be on the integer order of the wavelength λ of the wave generated. For most devices, the typical IDT spacing is a multiple of an integer or one half, one third, or one quarter of the wavelength λ=2π/k, where k is the wavenumber along the direction of the wave propagation. The acoustic wavelength can also be obtained using the equation λ=c/f, where c refers to the phase wave velocity and f refers to the central frequency of the surface wave. As the number of fingers (nf) increases, the aperture (W) decreases, the transmitted wave energy increases, and thus the wave amplitude increases. However, within one wavelength λ, if the number of fingers increases, the SAW sensor generates higher harmonics. By controlling the finger width (w) to period (L) ratio, the period L < λ controls the number of harmonics generated.

The interdigitated electrodes act as a capacitive system. As per the IDT configuration, the total capacitance is given by

(15) $$\;C_{total}=1/2\pi fZ$$

where Z refers to the impedance of the IDT. It has been shown that, for the best responsive result, the impedance of the IDT should match the impedance of the whole measurement system. Another important factor is the aperture (ap) of the IDTs. Considering the overlapped part (fingers) of the IDT system, the aperture W is given by

(16) $$\;W=C_{total}/C_{o}\cdot N$$

where N represents the total number of the IDT fingers.

For the sensing purpose of a SAW device, the central frequency and the insertion loss are also crucial. For example, a very sensitive SAW-based sensor must possess a central frequency in the GHz range. However, to achieve this, the system suffers insertion losses. Due to one of these reasons, there are various designs of IDTs that enhance efficiency, performance, and stability based on the application or the required system [133,134,135]. Following the discussion, the pros and cons of each IDT design are tabulated in Table 4.

### 4.1. Delay Line Configuration

**Figure 9:**
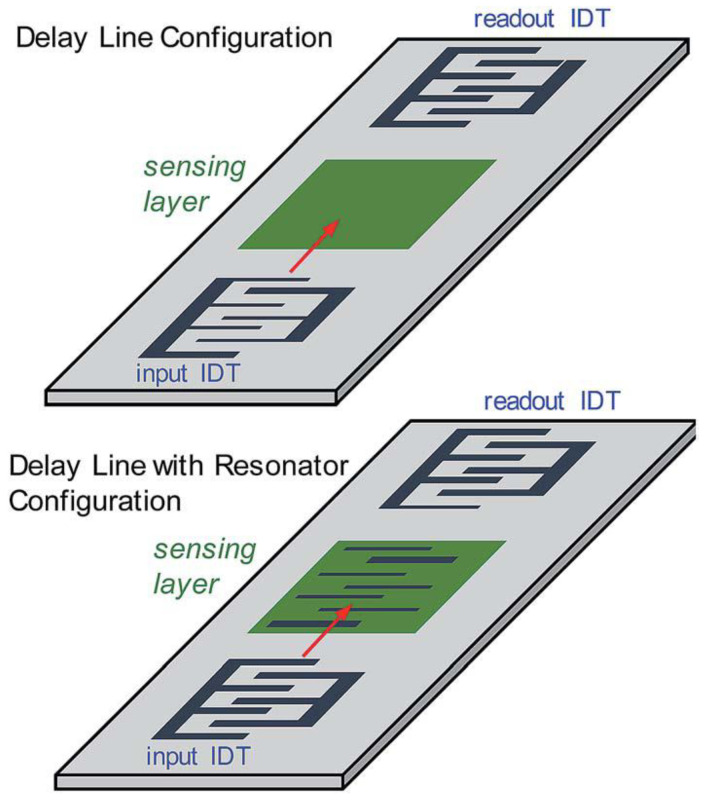
The delay line configuration setup with and without resonator [17].

As for the SAW devices, a single interdigitated electrode-based configuration can be used to generate planar traveling waves that propagate along the surface of the piezoelectric device. However, a set of two or more opposing interdigitated electrodes can be used to generate constructive interference and produce standing waves. One such design and the basic configuration is known as the delay line configuration. Figure 9 describes the setup of the basic delay line configuration with and without resonators.

The delay line configuration consists of two IDTs present on top of the piezoelectric substrate. The first IDT or the input IDT generates the surface acoustic waves, and the generated wave propagates through the sensing platform. The wave interacts and an electrical signal is generated on the second IDT, which is the readout, due to the piezoelectric phenomenon [136,137]. Basically, the distance between the readout IDT and the input IDT is called the delay line where, in general, the sensing layers are deposited. A very important parameter known as the Q factor or quality factor is often used in the fields of physics and engineering. The quality factor is a dimensionless parameter that indicates the energy losses in the resonant element. For electrical and RF components, the Q factor is the ratio of the energy stored in a resonator to the energy supplied to it per cycle. A Q factor with high values increases the resonance, which directly enhances the sensitivity and the selectivity of the sensor devices [138,139] defined at the end of Section 1. The amount of the energy losses is very low with a higher quality factor, causing the oscillators to oscillate with a compact range of frequencies and thus improving the stability of the devices. For SAW sensors, a special configuration exists in which a pair of additional electrodes is deposited to enhance the quality factor and decrease the insertion loss that reflects to produce resonating cavities. The resulting resonant cavity helps in focusing the acoustic wave energy to the substrate, which subsequently helps to improve the performance [140,141,142,143].

**Figure 10:**
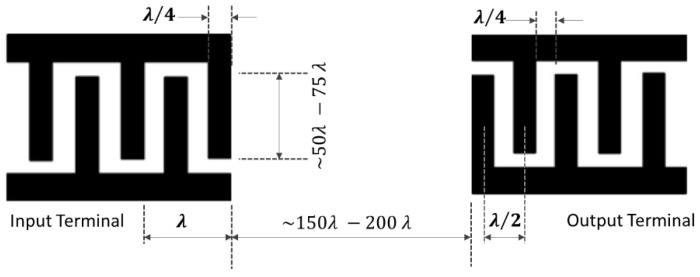
Schematic representation of the bi-directional electrodes [2].

The general delay line configuration or the bi-directional electrode has a finger spacing that usually consists of a grating structure with the intervals of λ/4. Figure 10 shows a typical layout of the bi-directional electrodes.

### 4.2. Split Electrodes or Double Electrode Configuration

**Figure 11:**
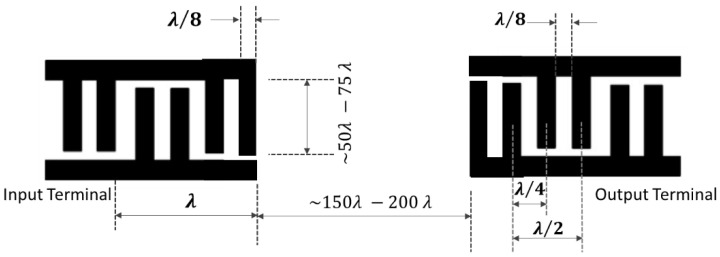
Schematic representation of the split electrode configuration [2].

The split electrode design consists of a grating structure that has the electrodes at intervals of λ/8 along with a center-to-center distance of λ/4. The primary reasons for using a split electrode transducer is to eliminate reflections, to minimize triple transit and self-resonance, and to operate at the third harmonic to access higher frequencies. Figure 11 describes the design of the split electrodes. The disadvantage of the split electrode configuration is that it is not as effective in the reduction of the reflection of the waves as intended. The split electrodes allow operation at the third harmonic, giving room for the higher frequencies for a specific minimum electrode width [2,90,144]. In the split electrode configuration, there exists a design, which is also known as the meander line, in which all the gaps and the electrode fingers are at 3λ/8, which produces a third harmonic response stronger than its original response.

### 4.3. Single Phase Unidirectional Transducer (SPUDT) Electrode Configuration

**Figure 12:**
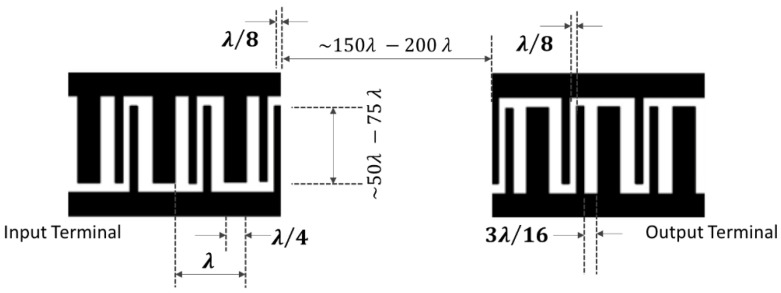
Schematic representation of the SPUDT electrode configuration [2].

The single-phase unidirectional transducer is another configuration consisting of interdigitated that which nullifies the regenerated waves with internally configured reflectors and thus generates unidirectional surface acoustic waves that propagate from one side of the IDTs. This special design has the potential to eliminate the triple transition effect and reduce the insertion loss. The single-phase unidirectional transducer design has an electrode finger width of λ/4 and λ/8 with an interval spacing of λ/8 and 3λ/16 [2,90,145,146] in both input and output IDTs. Figure 12 shows the design of the SPUDT. The SPUDT-based SAW devices have their application in microfluidics and sensors. The design has shown not only improved performance but also maintains the devices at the best operating conditions.

### 4.4. Distributed Acoustic Reflecting Transducer (DART) Configuration

**Figure 13:**
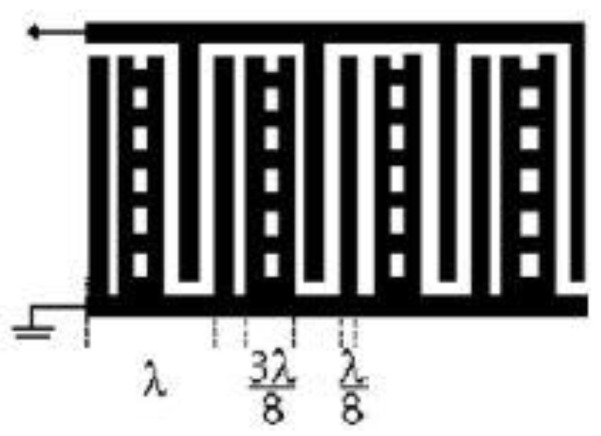
Schematic representation of the DART electrode configuration [2].

The distributed acoustic reflecting transducers (DARTs) consist of a series of similar cells with a length equivalent to the wavelength λ. In this design, each cell contains two electrodes with λ/8 as their width, with individual electrodes as λ/4. The cell consists of the inter-electrode region, which is equal to λ/8. Figure 13 shows the design of the DART configuration. In this configuration, different reflections can be attained to nullify the net reflection and transmission effects [2,90,147,148,149]. Like the SPUDTs, the distributed acoustic reflecting transducer-based SAW devices are also used for sensing and microfluidic applications.

### 4.5. Floating Electrode Unidirectional Transducers (FEUDTs) Configuration

**Figure 14:**
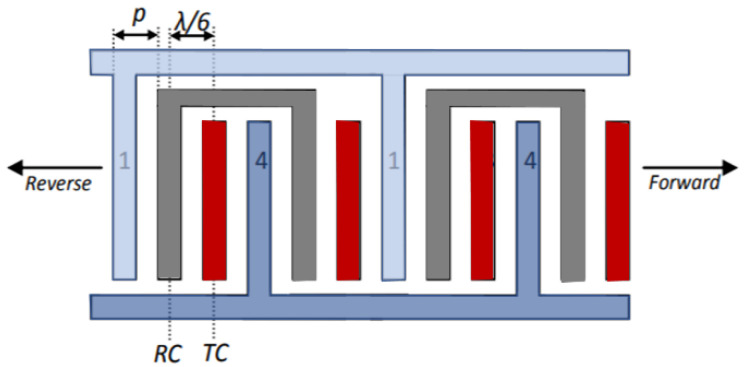
Schematic representation of the FEUDT electrodes configuration, where electrodes are numbered, 1, 2, 3, 4 designated by light blue, grey, red and dark blue colors, respectively in FEUDT layout [2].

One of the other electrode designs is the floating electrode unidirectional transducers. In this configuration, one or more electrodes are patterned and not connected between the simple interdigitated configurations. The electrodes appear to be freely floating as they are not connected to any subsequent terminals. The FEUDTs have the advantage of producing higher frequencies from the same electrode width when compared to the conventional interdigitated pattern. It is reported that the FEUDTs type of pattern consists of higher acoustic energies in the forward directions and lower insertion losses when compared to the bi-directional IDTs [2,90,150,151,152]. This phenomenon directly results in better sensitivity. The FEUDTs are also widely used in sensors and microfluidics where higher operational frequencies are required. Figure 14 shows the FEUDTs configuration.

### 4.6. Dispersive Delay Line (Chirped) Electrode Configuration

**Figure 15:**
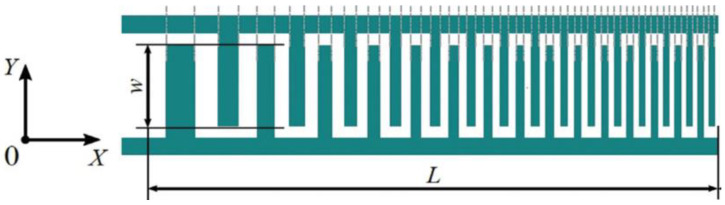
Schematic representation of the dispersive delay line electrode configuration [153].

The dispersive delay line is a type of IDT design for SAW devices in which the fingers of the electrodes have varied dimensions. The pitch and the width of the electrodes have different values within the same unit of the whole IDTs. The dispersive delay lines have varying widths that produce altered frequencies in the electrodes. The varied frequency setup enables the wave modes and the reflectivity to be controlled and for the pitch of the wave to be linearly modulated. This type of design can possess a huge number of bandwidths [2,90,153]. Figure 15 shows an example of a dispersive delay line configuration. This design can be used for microfluidics and other biochemical sensing purposes. It was found that the dispersive delay line design can also be used for the manipulation of the droplets in fluid dynamics, and it helps in producing focused acoustic energies.

### 4.7. Tapered Interdigitated Electrode Configuration

**Figure 16:**
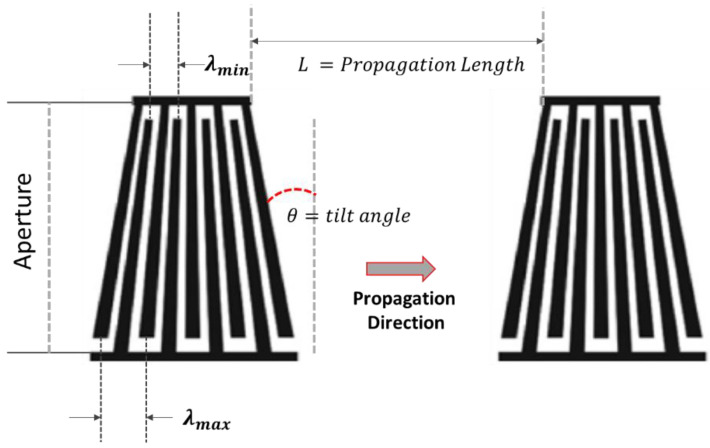
Schematic representation of the tapered electrode configuration [2].

The tapered or slant interdigitated electrode configuration has a similar concept to that of the dispersive delay line configuration. This design consists of each individual electrode finger, which is tapered. The width of each electrode finger gradually changes from thicker to thinner and vice versa. Like the dispersive delay configuration, the tapered IDTs also enable varying frequencies to be produced with the electrodes due to the gradual change in the periodicity. This configuration allows the system to achieve a broader bandwidth. The slant electrode design is widely used in various applications, such as the non-destructive evaluation of the thin films and measurements of the band gaps in the phononic crystals [2,90,154,155,156,157,158]. Figure 16 denotes the schematic of the slant interdigitated electrodes.

### 4.8. Focused Electrodes Configuration

**Figure 17:**
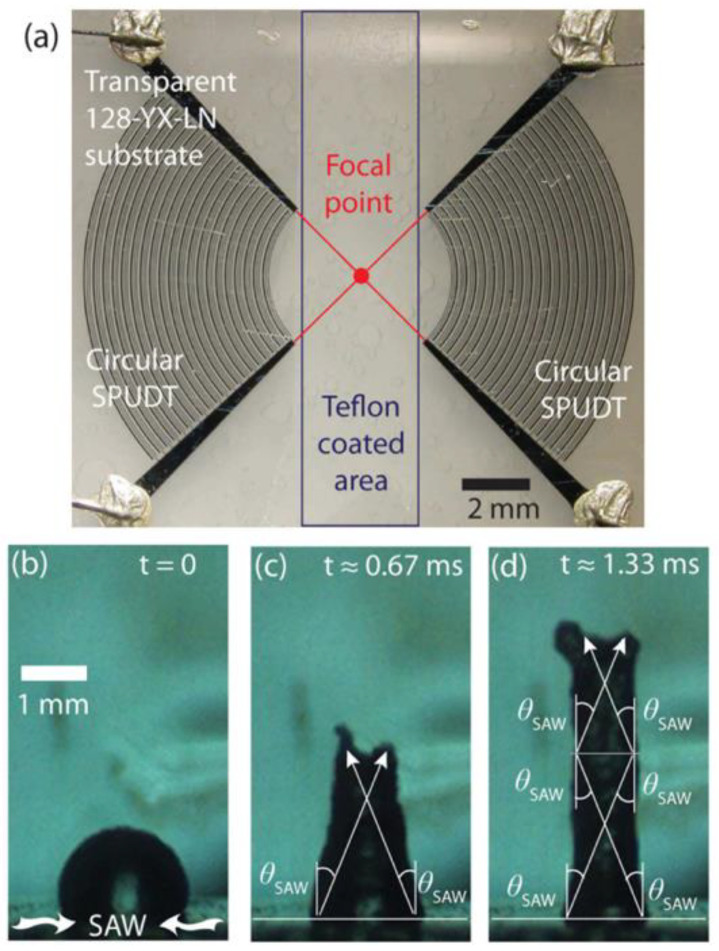
Focused electrode configuration and F-SAW on liquid manipulation [77]. (**a**) a droplet is placed into the directed area on which the focused SAWs radiate into the droplet. Jet formation of the droplet at time (**b**) t = 0 s, (**c**) t = 0.67 ms and (**d**) t = 1.33 ms due to focused SAW exposure.

The focused electrodes are a type of design of the IDTs in which the interdigitated electrodes are designed or curled in a particular manner such that all the generated waves propagate to a particular point or focus of interest. Due to this typical circular design, a large amount of focused acoustic energies is generated. This acoustic energy is used for better pumping and mixing, particularly in the field of fluidics. Additionally, due to the focused waves phenomenon, higher sensitivity is reported in the case of the sensors as more wavefronts passes through a small area of interest [2,90,159,160,161,162,163]. Tan et al. (2009) [77] performed an experiment in which the superposition of the focused surface acoustic waves produced a strong standing wave. A droplet is placed into the directed area on which the focused SAWs (Figure 17a) radiate into the droplet. Due to this phenomenon, the droplet placed in the piezoelectric substrate deforms into a coherent elongated jet. Figure 17 shows the schematic of the circular electrodes and the jet formation of the droplet. This configuration is widely used in both sensing and actuation applications. Additionally, this design is reported to take advantage of a higher signal-to-noise ratio that enhances the sensitivity of a particular sensor.

When designing a particular set of interdigitated electrodes, different crucial parameters are taken into account such as the resonant frequency, power output density, the material of the electrodes, the geometry or dimensions of the electrodes, the properties of the piezoelectric substrate, and the quantity of reflective electrodes. Overall, studies were conducted and new configurations were developed to increase the efficacy of the acoustic waves, to enhance the spurious signal suppression, to reduce the amount of insertion loss, and to decrease the signal distortion. The following table (Table 5) lists the pros and cons of each type of IDT discussed above.

### 4.9. Sensing Membranes for the SAW Sensors

As mentioned earlier, the SAW sensors are not only used in electronics, but their use is also growing rapidly in the field of chemical and biomedical sensing. These sensors for detection purposes are often used in liquid and gas media. Thus, the SAW sensor consists of a sensing film and a conversion element. The sensing membrane or the sensing film is the main core of the SAW sensor, especially in the field of biosensing and chemical sensing. The sensing layer acts as a bridge, where the selective analytes or the target molecules are adsorbed on the surface of the sensing membrane. As a result, the selective mass loading of the analytes corresponds to the shifts in frequency, phase, or acoustic velocity that lead to the detection process. For these purposes, the selection of a highly sensitive and selective sensing membrane is desired for the SAW sensors. Although a wide range of materials is used as sensing membranes, these membranes are majorly categorized into polymers, semiconductors, and graphene-based sensing films [164]. The selection of a suitable sensing membrane involves several factors such as good selectivity of the target molecules or analytes, high response, stability, low-cost, and most of all, good biocompatibility with low toxicity [165]. SH-SAWs are mostly used for microfluidic, biological, and chemical sensing because of their excellent efficacy and low damping in liquid or gas media. The commonly used sensing membranes for this type of sensors are silicon dioxide and PMMA. Metal oxides have been widely used as sensing membranes over the years. These are known to be used for SAW sensing, especially gas sensing, because of their higher thermal stability, which makes them appropriate even under harsh conditions. Metal oxides such as silicon dioxide (SiO_2_), indium oxide (InO_2_), tungsten trioxide (WO_3_), and zinc oxide (ZnO) are very commonly utilized as sensing membranes [165]. In addition, cobalt oxide (Co_3_O_4_) has been reported as a sensing membrane for the detection of the carbon monoxide, hydrogen, ethanol, and nitrogen. Polymers are another excellent class of sensing membrane materials that are extensively utilized in SAW sensors. Polymers possess the advantage of easy integration into the transducer of the SAW sensors and better efficiency under room temperature. The polymers that are promising and most commonly used as sensing membranes are polyacetylene, polythiophene, polypyrrole, polyaniline, etc. [165]. The chemical molecules or analytes upon adsorption on the surface of this polymeric-based membrane correspond to the shift of the modulus of the polymers due to the viscoelastic effect. As a result, the velocity of the wave is affected, which also influences the change in the frequency. Many other chemoselective polymers are reported, such as polyethylene imine (PEI), poly(epichlorhydrin) (PECH), polyisoprene (PIP), polybutadiene (PBD), etc., which are used as sensing membranes, especially for the detection of chemical warfare agents [166]. Similarly, there are many other materials such as graphene and carbon nanotubes that are widely employed as a sensing membrane for SAW sensors. The choice of sensing membranes for SAW sensors in itself is a vast and open field of research with advancements in different directions under different physical and chemical conditions. This section demands a complete individual review paper in its own right, and hence detail descriptions are omitted herein.

## 5. SAW Applications

### 5.1. Biosensing Applications

**Figure 18:**
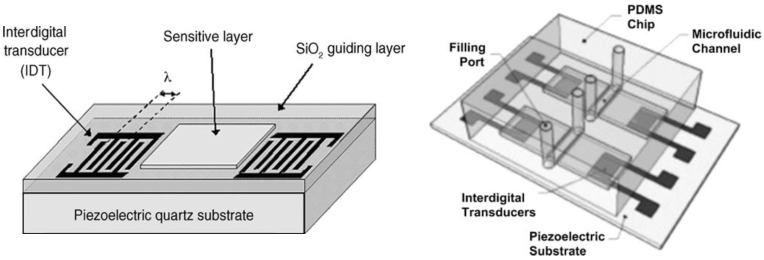
Love-wave sensing platform with PDMS microchannels for biosensing [167].

Biosensing employing SAW technology has had its most significant applications in recent times. The interest in this field has grown with a particular focus on separating, identifying, and controlling biological targets such as biomolecules and/or proteins from bio-species such as bacteria, fungi, viruses, etc. Figure 18 [167] shows the schematic of the most generic form of the Love wave-based biosensor with the PDMS microchannels.

In general, the antibodies are functionalized over the SAW sensing area, and the analytes that bind with these specific antibodies or targets form a conjugate bio-complex that then perturbs the acoustic phase velocity. These changes or perturbations are then measured using the IDTs in the SAW devices followed by a mathematical analysis using the software. This is the fundamental mechanism of biosensing using SAW devices [168,169].

**Figure 19:**
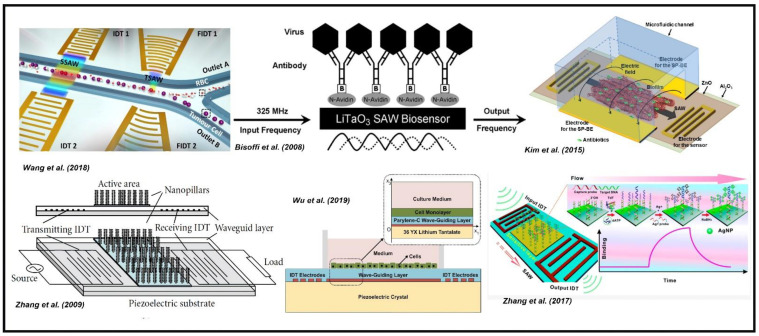
Using a similar philosophy, different applications of Love-wave based SAW sensing where input frequencies and output frequencies are compared to detect the change in the bio-functionalized layer [171,172,173,174,175,176].

Figure 19 shows the various SAW-based biosensors for different biological applications such as virus detection, cell separations, tendon stem cell monitoring, bacterial biofilms, DNA detection, etc. The derivatives of the basic approach are shown in Figure 19. All these different biological applications are performed using the generic configuration in which the bio-functionalized layer or the biosystems are placed between the two adjacent interdigitated electrodes, followed by exciting the surface acoustic waves for either sensing or actuation. In the case of sensing, the biomolecule (target) binds with the sensitive layer, inducing a mass loading effect, resulting in a disturbance in the acoustic wave propagation and decrementing the phase velocity and frequency modulation [170]. In contrast, in actuation—for example, cell separation—the acoustic waves are used to isolate different cells at a certain frequency due to the difference in mass and density of the cells. Wang et al. (2018) [171] used this approach for cell separation using an active SAW device, Bisoffi et al. (2008) [172] used it for virus detection, Kim et al. (2015) [173] used it for the evaluation of antibodies bonded to biofilm, Zhang et al. (2009) [174] used the technique for detecting analytes reacting with nano rods, Wu et al. (2019) [175] proposed to monitor the adhesion process of tendon stem cells, and Zhang et al. (2017) [176] proposed a similar process for the detection of target DNA. Together with the quality of both sensing and actuation, the SAW platform acts as a powerful device in the field of biosensing. These are the few selected examples indicated regarding the surface acoustic waves used for biosensing and bio-actuation. However, there are various other research works and experiments that have been carried out over the years using surface acoustic waves with different physics, biological targets, sensing, and experimental setups, making this an ongoing hot topic in the field of biology.

### 5.2. Chemical and Gas Sensing Applications

**Figure 20:**
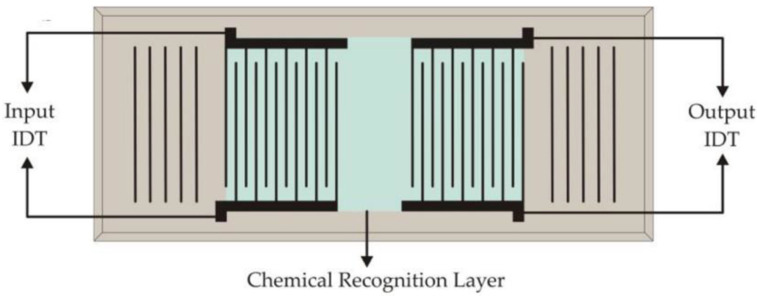
A fundamental schematic showing chemical and gas sensing applications using SAW devices [179].

Over the decades, SAW-based techniques have attracted a great deal of attention for chemical and gas sensing purposes. The basic principle of this type of sensing is quite similar to that of biosensing where a target chemical or gas molecule binds with the functionalized surface layer placed in between the two electrodes. The presence of the functionalized layer which binds with the target species results in perturbation to the localized material property and thus affects the acoustic wave. The change in the acoustic wave velocity affecting the frequencies is often measured. In case of chemical and gas sensing, the species mostly give rise to the change in the frequency. This change in frequency is studied in-depth and analyzed for the validation and quantification of effective gas sensing [177,178]. Figure 20 [179] represents a fundamental design of the gas/chemical-based SAW sensing platform.

**Figure 21:**
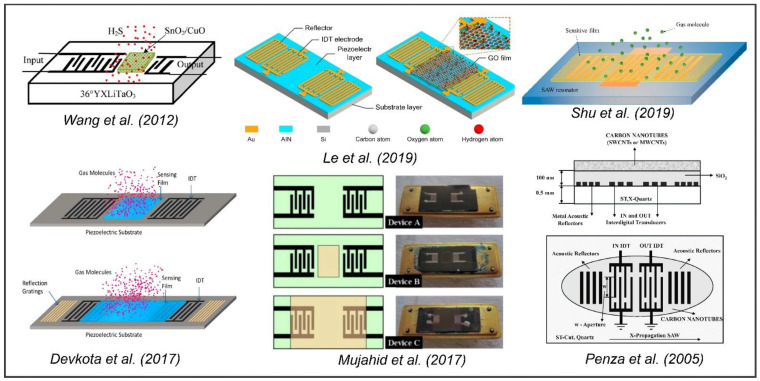
Different applications and methods of chemical and gas sensing using SAW devices [84,179,180,181,182,183].

As described in Figure 20, there are various other SAW sensing platforms that follow similar configurations but for different applications; for example, the detection of hydrogen sulfide by Wang et al. (2012) [180], or in a similar fashion, the detection of vapor, oxygen, and hydrogen gas, and even the detection of humidity by Le et al. (2019) [181], Devkota et al. (2017) [84], Shu et al. (2019) [182], Mujahid et al. (2017) [179], Penza et al. (2005) [183], etc. Figure 21 demonstrates a few different applications of SAW-based sensing for different chemical and gas sensing applications following the generalized configuration in Figure 20.

**Figure 22:**
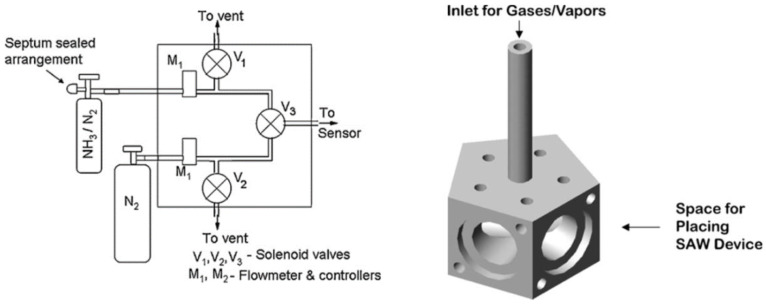
Schematic of the pentagon-shaped sensor cell and the gas delivery system [184].

These different applications of chemical and gas sensing are used based on the generic configuration, where the functionalized layer is either between or at the periphery of the interdigitated electrodes. The gas molecules (target) bind with the functionalized layer, inducing a mass loading effect and resulting in a disturbance in the acoustic wave propagation and decrementing the phase velocity determining the sensing process. There are also few unconventional designs that do not use the generic configuration. One of such experiments was conducted by Raj et al. (2010) [184], where the author used a gas delivery system in which the outlet system was connected to a pentagon-shaped five-channel SAW oscillator device for the detection of ammonia vapors. Figure 22 shows the schematic of the pentagon-shaped sensor cell and the gas delivery system.

The gas delivery system was designed in such a manner that one gas channel was connected to nitrogen and the other was connected to a mixture of nitrogen and ammonia at different concentrations. The gas delivery system consisted of a three-way solenoid valve to open one of the two paths, and its outlet was connected to the sensor directly. The four oscillators of the pentagon-shaped SAW sensor were coated with ZnO, and the fifth one was left bare for reference purposes. ST-X Quartz was used as the piezoelectric substrate for all the sensors, and the devices had the functionalization layer of ZnO depositions of 20 nm, 40 nm, 80 nm, and 100 nm, respectively. Different concentrations (0.25–25%) of the liquor ammonia were passed through the SAW sensors, and negative differential frequencies changes were observed corresponding to different adsorption and desorption rates of the ammonia vapors. These are a few selected examples describing surface acoustic waves used for chemical and gas sensing. Likewise, there are various other research works and experiments that have been carried out over the years and that are still on-going using surface acoustic waves with different experimental setups, functionalized layers, chemical targets, and different electrode configurations, corresponding to a powerful, promising device in the field of chemistry.

### 5.3. Microfluidics Applications

**Figure 23:**
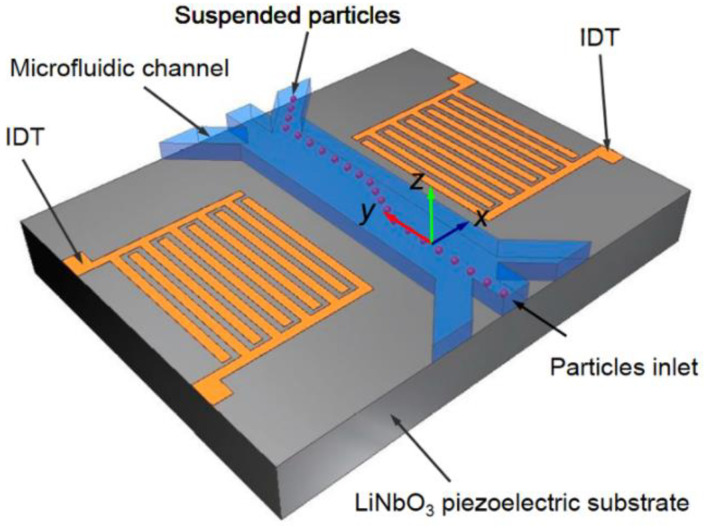
A generic approach for SAW devices with microfluidics [185].

As discussed earlier, SAW-based sensors are used for biological targets in the field of biosensing, and they can seamlessly interact in liquid media as well. The mechanism of interactions of the surface acoustic waves with liquid media can lead to phenomenal behavior that can not only be used for liquid sensing but also for the manipulation and mixing of the liquids in the sensing area. Surface acoustic waves have widely been used in the sector of microfluidics over decades. Applications such as microfluidic-based particle separation, the isolation of cells in a microfluidic platform, fluid manipulation, the atomization of droplets, mixing of microfluidics, fluid transport, and many other purposes are served with SAW-based phenomena. SAW-based technology is widely used by both sensors and actuators in the field of microfluidics, and thus a generic configuration of SAW-based sensing and actuation is discussed. In the field of sensing, certain functionalized particles are coated between the two adjacent interdigitated electrodes on top of the piezoelectric substrate. The sensing area is usually encapsulated with the microfluidic channels, and the fluid pertaining to the target particles to be sensed is passed along the microfluidic channel. The relevant interaction takes place in the presence of the surface acoustic waves generated by the piezoelectric substrate and analysis are made. Figure 23 [185] shows the schematic of a generic microfluidic-based sensing platform.

**Figure 24:**
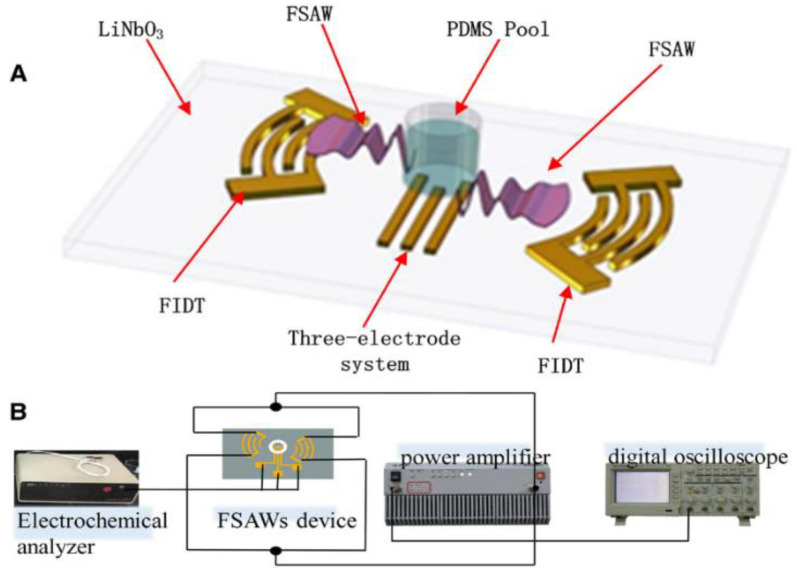

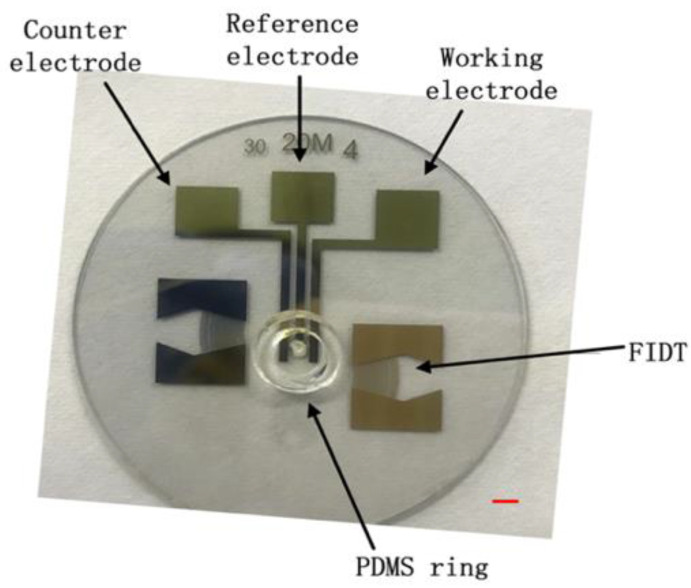
(**A**) FSAW platform for microfluidic mixing and (**B**) experimental setup [191].

Usually, in microfluidics, it is an intensive task to achieve the quick and efficient mixing of reagents. Various types of perturbations such as electrohydrodynamics, magnetohydrodynamics, and pressure have been utilized over the years to achieve this task [186,187,188,189,190]. However, this task is conveniently achieved using surface acoustic waves technology as an actuator. One of the generic configurations for SAW-based microfluidics is explained for fluid mixing (actuation). Figure 24 shows the schematic of the application of Focused Surface Acoustic Waves (FSAWs) in the field of microfluidics for enhancing the liquid mixing (actuation) and diffusion rate [191]. In this configuration, the microfluidic system is placed between the two focused interdigitated electrodes. The surface acoustic waves force the fluid into motion using low-intensity vibrations which allow the fluids to mix efficiently in an invasive way. Additionally, the acoustic energies are confined to the surface, which helps in generating the acoustic streaming [192,193].

**Figure 25:**
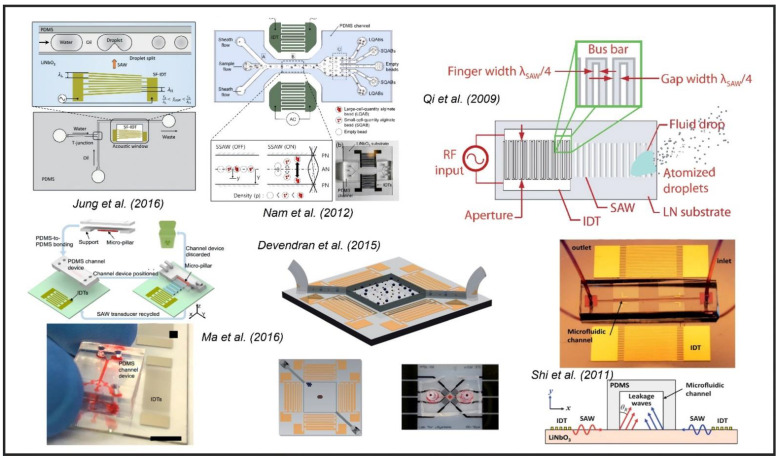
Various applications of SAW devices with microfluidics [194,195,196,197,198,199].

There are many other applications of SAW-based microfluidic devices that have been developed based on these generic configurations. Figure 25 shows different SAW-based microfluidic applications, such as droplet splitting by Jung et.al. (2016) [194], the separation of encapsulated cells by Nam et al. (2012) [195], the atomization of fluid particles by Qi et al. (2009) [196], and particle manipulation in microfluidics by Ma et al. (2016) [197], Devendran et al. (2015) [198], Shi et al. (2011) [199], etc. In summary, due to the properties of both sensing and actuation, a SAW-based device is powerful, and with time, it will be highly possible for the SAW devices to evolve beyond just a mere PZT crystal and maneuver into biomedical, chemical, and mechanical industries.

### 5.4. Mechano-Biological Applications

**Figure 26:**
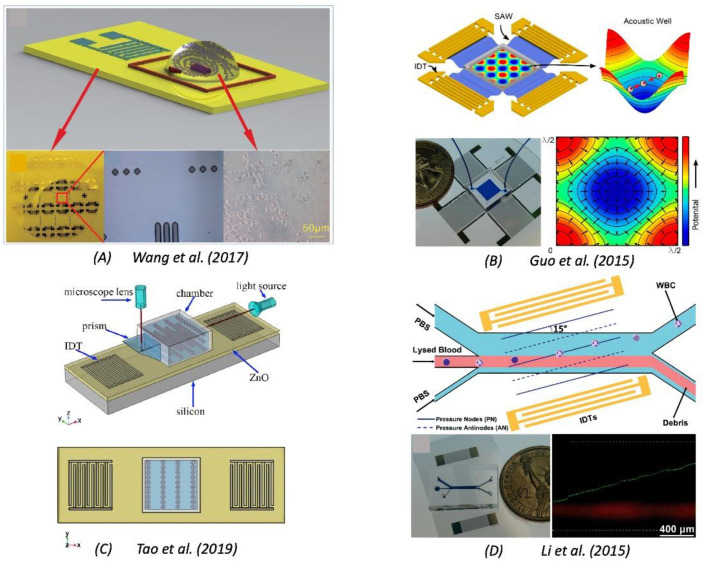
Various mechano-biological applications using SAW devices [37,200,201,202]. (**A**) SAWs for the purpose of cell lysis on a 128° YX-cut lithium niobate wafer, (**B**) controlling the cell–cell interactions by administering four orthogonal IDTs at a 45° angle to the X-axis of the 128 Y-cut lithium niobate substrate, (**C**) ZnO/Si thin films surface acoustic waves for the manipulating and 3D patterning of yeast cells and microparticles, (**D**) 15° tilted microfluidic channel with interdigitated electrodes on a 128° YX-cut lithium niobate wafer to generate a standing SAW.

As discussed earlier, SAW-based devices can not only be utilized for sensing purposes but also as an actuator. Unlike the sensors, the actuators consist of single or multiple pairs of interdigitated electrodes for the purpose of the manipulation of the target material. As discussed in Section 5.3, SAW-based devices are widely used in the microfluidics sector to manipulate the fluid in the microchannel using the surface acoustic waves. In a similar manner, there are different mechano-biological applications in which the SAW is used as an actuator. The surface acoustic waves actively interact with the biological particles and thus are manipulated by the user based on the designed IDTs, the input voltage, and other factors such as the type of substrate, distance between the target and IDTs, etc. The surface acoustic waves are actively used for wide mechano-bio purposes such as cell analysis, cell lysis, acousto-mechanical phenotyping, assembling of tissues, cell washing, isolation of the extracellular vesicles, sorting and patterning of the cells, etc. Li et al. (2015) [200] utilized a 15° tilted microfluidic channel with interdigitated electrodes on a 128° YX-cut lithium niobate wafer to generate a standing SAW resulting in the periodic distribution of the pressure nodes and antinodes inside the microchannel. The lysed blood samples were passed and the WBCs were washed based on the optimization of the frequency and the input voltage. Guo et al. (2015) [37] performed the action of controlling the cell–cell interactions by administering four orthogonal IDTs at a 45° angle to the X-axis of the 128 Y-cut lithium niobate substrate. Orthogonal standing SAWs were generated, and their superimposition produced the square pressure nodes (acoustic well). Modulating the input RF signals, the clusters of cells were trapped in the acoustic well, resulting in controlled cell–cell interactions. Similarly, Tao et al. (2019) [201] employed the ZnO/Si thin films surface acoustic waves for the manipulating and 3D patterning of yeast cells and microparticles. Wang et al. (2017) [202] utilized SAWs for the purpose of cell lysis on a 128° YX-cut lithium niobate wafer. IDTs were fabricated on top of the wafer, and a fluidic chamber was constructed where multiple micropillars of SU-8 photoresists were placed for the lysis purpose. Figure 26 shows the above application in four panels. The SAW generated using the RF power amplifier on the IDTs and the propagated surface acoustic waves induced high acoustic streaming, which forced the cells in the droplet placed in the fluidic chamber to impact with the micropillars, resulting in the destruction of the cell membranes and leading to the lysis phenomenon. The lysis rate and efficiency were both controlled by the amount of RF powers induced. Figure 26 shows the various applications of the SAW utilized for mechano-biological applications.

Additionally, studies were also conducted in which the separation of the particles, separation of the cells and the particles, and many more tasks are accomplished using the phase modulation-based SAW techniques [203,204,205]. The phase-modulation techniques are often used in SSAW devices, which create pressure nodes based on the constructive and destructive interferences (crests and troughs). The nodes are often utilized to separate the particles. Lee et al. (2017) [205] utilized the phase-modulation technique to separate human keratinocyte cells from a bead mixture with the advancement of continuous phase-modulated SSAW applied for the separation of the particles and cells in a micro-channel traversing multiple pressure nodes and enhancing the throughput. The technique utilizes the pressure nodes that are constantly displaced by the modulation of the phase of the acoustic waves to the rate for exerting the maximum acoustic radiation force on the particles. The drag force on the particles in the microchannels and the acoustic radiation forces balances, due to which the displacement occurs at a constant velocity equal to the pressure nodes. Thus, the continuous phase modulation results in the displacement of the particles of specific sizes and dimensions to the direction of the moving pressure nodes, while the non-target particles (of non-specific sizes) remain intact in the channel, causing successful separation; in other words, a successful mechano-biological application is achieved. Much other research works have been conducted in a similar fashion over the years and are still ongoing where the surface acoustic waves are employed for mechano-biological applications.

### 5.5. Possible Research Roadmap

SAW sensors will make a tremendous contribution to the growth of the biosensors industry in the future. However, to create this big leap, a few bottleneck problems must be solved. Currently, high selectivity and sensitivity of biosensors using the SAW devices discussed in Section 1 are unattainable simultaneously. Ensuring the simultaneous attainability of high sensitivity and selectivity will help SAW sensors to detect trace amounts of analyte confidently without false positives or false negatives. For microfluidic applications, it is necessary for the reaction and detection to be explored under water. However, Rayleigh waves in SAW sensors significantly attenuate due to the presence of water on the top surface. More research and energy should be spent to find better materials or mechanisms to explore SAWs under water such that both specificity and sensitivity can be enhanced. Most SAW sensors are devices designed for very high frequencies >50 MHz. This requires a special fabrication process. However, low frequency applications of SAWs between 1–5 MHz are required to be explored for biosensing, chemical and gas sensing, and microfluidic applications. Materials for biosensors should be carefully selected to be safe for bio testing. Hence, biocompatible SAW sensor materials should be used. More research is required to develop a novel biocompatible piezoelectric substrate that will help in the safe implementation of SAW sensors for bio applications. Currently, the main drawbacks of SAW sensors are that they are mostly research devices created in laboratories for feasibility studies and they are expensive. Not many SAW bio sensors have matured as a product for mass scale implementation. In order to create cheaper and safer SAW sensors as a product, a standard design of IDTs and a standard and safer material type should be used to create devices in micro fabrication facilities on a mass scale. The most achievable product-driven application would be SAW sensors that use a shear-horizontal wave with a top analyte coating generating Love waves.

## 6. Conclusions

It is highly evident from the above presentation that SAW-based techniques are in high demand and an active research area because of their potential application to different engineering, biological, and chemical applications as sensors and actuators. SAW devices could be low cost, small, and consume low power. SAW devices are very trustworthy and compatible even under different unfavorable ambiances such as structures with high temperatures and magnetic fields. Even though there are a wide variety of applications of these devices and advantages, there are many opportunities where improvements can be made for future SAW sensors. SAW sensors, especially for biological and chemical/microfluidic-based sensing, at times suffer the limitation of the damping effect, especially in liquid media. Efficacy with higher sensitivity and specificity under the same sensing platform is often challenging. The high cost of packaging and the difficulties in integrating microfluidics make these devices less attractive compared to other commercially available devices for diagnostics and sensing. It is also seen that the flexible polymeric SAW-based sensors are a promising technology, but they also suffer challenges in the fabrication and manufacturing processes, such as controlling the film thickness, the dispersion, and the damping of the SAW on the polymeric substrate, improper adherence of the piezo film to the polymeric substate etc. In the field of microfluidics, quantitative analysis of the non-linear interactions between the liquid and particles on the SAW devices has much scope for improvements. Although studies are on-going on these challenges, these areas can be further explored to over these obstacles and advance the devices for better commercialization. Several biosensors and other chemical sensors have used smartphone technologies to facilitate the access to rapid and cost-effective diagnostic platforms. Although a few investigations have been performed, a substantial exploration of the integration of smartphone technology with SAW-based sensors for detection will be immensely valuable, especially in rural areas and low-income countries.

In this article, we have attempted to present the wide variety of techniques used in SAW techniques, the state of the art, and applications in different fields—mainly biological and chemical, microfluidics, and mechano-biological—over the course of their history. We have also presented how this field and SAW devices could impact our technological advancement in the upcoming future. The article discusses the factors that affect sensing and the types of materials and waves associated with the SAW techniques. With the help of appropriate knowledge on the design parameters and the selection of specific materials, a SAW-based device can be developed to achieve effective sensing and actuation needs. It is evident that the present and long-term evolution of SAW-based techniques will impact various scientific research communities and will be an exciting field to follow. With the ongoing desperate need for highly sensitive, selective, and more financially effective technologies for point of care devices, SAW-based techniques have a huge potential and have a great chance to make major incursions and become a crucial player in the field of technology and public health.

## Figures and Tables

**Table 1:** Table describing types of waves and materials to be used.

Wave Type	Materials	Applicable Frequency Range	Attenuat ion	Misc. Comments
Rayleigh Wave	128° YX lithium niobate 41° YX lithium niobate ST-Quartz	~3 MHz–~2 GHz Most application 345 MHz	Highly attenuative With fluid load on the top of the substrate, attenuation is significant	For Rayleigh wave generation, power consumption is lower Can be of low cost
Lamb Wave	Generated in any bounded plate-like structure. This wave is generated in layered media at lower frequencies. However, for high frequency applications, it is hard to find natural material where Lamb wave could be used for SAW sensors. Thus, artificial thin films—e.g., ZnO, AlN, PVDF thin film—could be used	~200–~2 MHz in natural materials 200 MHz–2 GHz in artificially made thin films	Low attenuation, propagates long distance In presence of liquid load on the substrate, leaky-Lamb wave propagates Leaky waves attenuate faster	Lamb waves consists of Antisymmetric and Symmetric wave modes. First antisymmetric ($A_{0}$) and first symmetric ($S_{0}$) wave modes are easy to detect with appropriate delay lines.
**SH-Wave**	64° YX lithium niobate 36° YX lithium niobate Quartz	~100–~450 MHz	Nondispersive Low attenuation; however, sometimes combined with bulk wave, so hard to detect	Low cost Wide application, Suitable to use with fluid loading on the substrate
Love Wave	Must have a guiding layer on top of the substrate Possible substrates: Similar to SH-wave Possible guiding layers: SiO_2,_ ZnO, TiO_2_, SU-8 photoresists, polymethyl methacrylate etc.	~100–~450 MHz same as SH wave	With increasing coating thickness insertion loss increases Attenuation in guiding layer or the coating layer affects significantly	Highly sensitive Works in fluid loading environment

**Table 2:** Comparison table for the properties of different bulk piezoelectric materials.

Materials	Orientation/Cut	Piezoelectric Coefficient (C/N), d33	Wave Velocity (m/s)	Density (Kg/m^3^)
Quartz	X axis	2.3 × 10^−12^ (d11)	3159 (Transverse)	2650
Quartz	ST-cut	2.3 × 10^−12^ (d11)	3159 (Transverse)	2650
Lithium niobate	Y, Z axis	6 × 10^−12^	3488	4650
Lithium niobate	128° Y, X axis	12 × 10^−12^	3992	4650
Lithium tantalate	Y, Z axis	8 × 10^−12^	3230	7465
Lithium tantalate	X-cut	5.7 × 10^−12^	3290	7465
Langasite		7 × 10^−12^	2723 (0°; 138,5°; 26,7°)	5746
PVDF film		20 × 10^−12^	2600 (Longitudinal)	1780
Lead zirconate titanate (PZT)		110 × 10^−12^	3900 (Longitudinal)	7500
Barium titanate		160 × 10^−12^	4392	5700
**Materials**	**Orientation/Cut**	**Elastic Modulus (GPa)**	**Curie Temperature (°C)**	**Electromechanical Coupling Coefficient, k^2^ (%)**
Quartz	X axis	97.2	573	0.14
Quartz	ST-cut	71.7	573	0.03
lithium niobate	Y, Z axis	202 (C_11_)	1150	0.045
lithium niobate	128° Y, X axis	202 (C_11_)	1150	5–11.3
Lithium tantalate	Y, Z axis	233.1 (C_11_)	607	0.66
Lithium tantalate	X-cut	233.1 (C_11_)	607	0.75
Langasite		189.2 (C_11_)	N/A	0.36
PVDF film		2.5	80–100	2.9
Lead zirconate titanate (PZT)		60	200	20–35
Barium titanate		110	120	0.34

**Table 3:** Description of different piezoelectric materials and their cuts for specific wave generation.

Bare Substrates (Unless Specified with Thin Film)	Direction of Wave Propagation	Rayleigh Waves	SH-Waves	Love Waves
128°YX lithium niobate	X-direction	√		
64° YX lithium niobate, with coating	X-direction			√
36° YX lithium tantalate	X-direction		√	
36° YX lithium tantalate	Y-direction	√		
36° YX lithium tantalate with coating	Y-direction			√
YZ-cut lithium niobate	Z-direction	√		
Langasite	Euler angle (0°, 22°, 90°)		√	
Potassium Niobate	Euler angle (0°, 90°, 0°)		√	
ST-cut Quartz with PMMA or SiO_2_ layer	Y-direction			√
41° YX lithium niobate	X-direction		√	
36° YX lithium tantalate with PMMA or SiO_2_ layer	X-direction			√
ZnO thin films	Euler angle (90°, 90°, 0°)	√		

**Table 4:** Description of different deposited piezoelectrical material properties.

Properties	ZnO	GaN	AlN	GaAs
Density (Kg/m^3^)	5610	6150	3300	5317
Elastic modulus (GPa)	140	320	300–350	86
Poisson’s ratio	0.36	0.183	0.29	0.31
Refractive index	2	2.5	1.96	3.85
Piezoelectric coefficient (pC/N), d33	12	4.5	4.5, 6.4	3.4
Electromechanical coupling coefficient, k^2^ (%)	1.5	0.13	3.1–8	0.07
Wave velocity (m/s)	2720	4130	5800	4730
Dielectric constant	8.66	8.9	8.5–10	12.9
Coefficient of thermal expansion (/°C)	6.5 × 10^−6^	3.17 × 10^−6^	5.2 × 10^−6^	5.73 × 10^−6^

**Table 5:** Comparison and recommendations of the IDT types for SAW applications.

Electrodes Configuration	Pitch and Electrodes Distances	Advantages	Disadvantages	Recommended Applications
Delay line configuration	λ/4	Simple design. Basic IDT structure easy to fabricate	Internal mechanical edge reflections, loss of wave energy, low quality factor	General basic SAW usage
Split electrode configuration	Electrodes—λ/8 Center to center—λ/4	The operation at third harmonic is feasible allowing higher frequency for a minimum electrode width	The efficiency of reducing the reflection is not enough	General basic SAW usage
Single phase unidirectional transducers (SPUDT)	Electrode width—λ/4 and λ/8 Interval spacing—λ/8 and 3λ/16	Reduce insertion loss. Cancels the regenerated waves using the internal tuned reflectors	Reduction in total SAW energy	Microfluidics and sensors, mobile and radio applications, gas sensing
Distributed Acoustic Reflecting Transducers (DART)	Each cell distance—λ Each cell consists of two electrodes—λ/8 one electrode—λ/4	The DART configuration possesses the advantage of cancelling the net reflection and the transmission effects that it achieves using variable reflection	The fabrication of the electrodes is slightly complicated comparatively	Sensors and actuators, microfluidics and other general SAW-based application
Floating Electrodes Unidirectional Transducers (FEUDTs)	Floating electrodes to the terminal electrodes—λ/6	Produces higher frequency from the same electrode width compared to conventional IDT. Higher acoustic energies and lower insertion loss	The fabrication of the electrodes is slightly complicated and not recommended for applications with a low desired frequency	MEMS applications, sensors and actuators with highly desired frequency
Dispersive delay line configuration	The pitch and the electrodes thickness decrease gradually as per the user configuration or the requirement	The varying width and the pitch help in producing different frequencies range over entire electrode. A large number of bandwidths are attained	The fabrication is slightly more complicated than the conventional IDT	MEMS applications, sensors and actuators, microfluidic manipulation, chemical and biosensing etc.
Tapered interdigitated electrodes	The width and the pitch of the electrode changes from thicker to thinner and vice-versa as per the user.	The tapered IDTs produce varying frequencies with the electrodes as the periodicity is changing. A broader bandwidth is achievable	The fabrication is slightly more complicated than the conventional IDT	Non-Destructive Evaluation of thin films, phononic crystals band gaps measurements, MEMS applications, sensors and actuators, chemical and biosensing, mechanical sensing etc.
Focused electrodes	The electrodes are configured in a circular arc with an increasing radius along with uniform width and thickness	The focused electrodes focus high number of acoustic energies concentrated to a particular point of interest	Not suitable for the applications where strong acoustic energies are not desired. Highly specific and restricted to shorter area of interest	Mechanical/microfluidics pumping and mixing. Suitable of better actuation and resolution in the field of biomedical, chemical, and mechanical applications based on SAW
